# Swine influenza surveillance in Italy uncovers regional and farm-based genetic clustering

**DOI:** 10.3389/fmicb.2025.1607204

**Published:** 2025-07-21

**Authors:** L. Cavicchio, L. Tassoni, A. Pastori, M. Carrino, L. Gagliazzo, M. Mion, Martina Ustulin, D. Vio, C. Mantovani, L. Ceglie, A. Fusaro, M. S. Beato

**Affiliations:** ^1^Diagnostic Virology Laboratory, Istituto Zooprofilattico Sperimentale delle Venezie (IZSVe), Padova, Italy; ^2^Department of Comparative Biomedical Sciences, Istituto Zooprofilattico Sperimentale delle Venezie (IZSVe), Padova, Italy; ^3^Department of Epidemiology, Istituto Zooprofilattico Sperimentale delle Venezie (IZSVe), Padova, Italy; ^4^Pathology and Welfare Laboratory of Swine Species, Istituto Zooprofilattico Sperimentale delle Venezie (IZSVe), Padova, Italy; ^5^Science Communication Laboratory, Istituto Zooprofilattico Sperimentale delle Venezie (IZSVe), Padova, Italy

**Keywords:** swine influenza, genotype, diversity, Italy, antigenic

## Abstract

**Introduction:**

Swine Influenza is a respiratory disease endemic in pigs with implications for animal and public health. Pigs, as mixing vessels for human, avian, and swine influenza strains, contribute to viral reassortment and emergence of new strains. Influenza viruses can circulate and spread unnoticed between pig farms for extended periods, heightening the risk of reassortment events. This study aimed to monitor swine Influenza virus (swIAV) genetic diversity in Northern Italy and understand its evolution in the study area.

**Material and methods:**

Passive surveillance, conducted from 2013 to 2022, involved 253 farms located in three regions, collecting over 3,000 samples that were tested for swIAV. Eighty-five samples underwent full genome sequencing, and phylogenetic analyses were conducted for each segment. In addition, cross-reactivity of viral strains was assessed using hemagglutination inhibition (HI) tests with hyperimmune swine sera.

**Results:**

Of the tested farms, 37.9% were positive for swIAV on at least one sampling event. Twelve distinct genotypes were identified, including two novel genotypes in Italy, both detected in 2022. Phylogenetic analyses revealed the presence of strictly correlated viruses in farms sharing the same owner or geographical proximity and highlighted multiple introductions and reassortment events in some farms. Cross HI tests demonstrated minimal antigenic cross-reactivity between circulating swIAV strains.

**Conclusion:**

The study reveals a high genetic diversity in swIAV circulating in Northern Italy as a consequence of multiple virus introductions as well as new reassortment events with the identification of two new genotypes. The findings highlight the importance of sustained surveillance and genetic monitoring to track viral evolution and reassortment, which are pivotal for early detection of strains with pandemic potential.

## 1 Introduction

Swine influenza is a frequent viral respiratory disease of swine caused by Alphainfluenzavirus belonging to the *Orthomyxoviridae* family (Rovid Spickler, 2009) commonly defined as influenza type A virus (IAV). The clinical manifestations in pigs may vary according to age, animal health and vaccination status, maternal immunity and virus strains (Torremorell et al., 2012). IAV is an RNA negative-sense, single-stranded and eight-segmented virus with two genes encoding two main antigenic surface glycoproteins: the hemagglutinin (HA) and neuraminidase (NA), and the remaining six gene segments encode internal proteins, namely polymerase B2 (PB2), polymerase B1 (PB1), polymerase A (PA), nucleoprotein (NP), matrix proteins (M1 and M2), and non-structural protein (NS) (Abdelwhab and Mettenleiter, 2023). The segmented nature of the virus provides the basis for genetic reassortment with the generation of virus strains with a new gene constellation (Abdelwhab and Mettenleiter, 2023). Pigs are considered potential mixing vessel for reassortment of influenza viruses from different host species (Li and Robertson, 2021). They display a dual susceptibility to avian and mammalian influenza strains for the virus-binding sialic acid receptors (α-2,6 and α-2,3) which facilitate infection and transmission from different hosts (Ito et al., 1998). Endemic (Spackman and Sitaras, 2020) swine influenza A strains (swIAV) are commonly differentiated into four principal subtypes: avian-like H1N1, human-like H1N2 and human-like H3N2 and the pandemic H1N1 (H1pdmN1) (Lewis et al., 2016).

Until 1979 the “classical swine” (CS) H1N1 virus, originated from the 1918 Spanish flu pandemic, was the only lineage circulating in European swineherds (Anderson et al., 2021). The CS virus was replaced by an avian originated strain detected in 1979 in Belgium, defined as “avian-like H1N1 (H1avN1)” or Eurasian avian-like H1N1” (EA) (Anderson et al., 2021; Brown, 2000; Zell et al., 2013).

The EA H1avN1 became predominant in Europe, and through reassortment with a human seasonal IAV, and human like swine H3N2 strains, generated the human-like H1N2 (H1huN2) with A/swine/Scotland/410440/94 being the progenitor (Brown, 2000; Lam et al., 2008; Watson et al., 2015). The H1pdmN1, firstly detected in humans in Mexico in 2009, originated from the reassortment event between the North America triple reassortant swIAV and the EA H1avN1 (Neumann et al., 2009; Smith et al., 2009). As early as September 2009 this lineage was detected in European swine (Welsh et al., 2010), and since then has become established in European countries at varying frequencies.

The most recent ancestor of H3N2 in European pigs originated from a 1970s human strain (Anderson et al., 2016, 2021; Straw, 2006), which in the 1980s mixed with the H1avN1 strain, acquiring its internal genes (Anderson et al., 2021; Castrucci et al., 1993). By the 1990s, a triple-reassortant H3N2 emerged, combining human, avian, and swine influenza genes, evolving into distinct clades that continue to circulate (Zhou et al., 1999). In the 2000s, a new H3N2 lineage was identified in Danish pigs, with the H3 gene of human origin, N2 from swine, and internal genes from the H1N1pdm09 virus (Krog et al., 2017).

However, due to the great genetic variability within each subtype, regional classifications were developed over time, adopting different *ad hoc* genotype nomenclatures.

With the purpose of harmonizing the classification system, swIAV are now classified based on the HA and NA genes phylogeny and the whole genome constellation. The most recent global HA classification discriminates the H1 into three lineages: A corresponding to strains highly related to the 1918 human influenza pandemic; B including all the H1hu strains, and lineage C including the EA H1avN1 strains; lineage is further divided into different clades and subclades (Anderson et al., 2016).

Swine H3 viruses are classified by the period when their ancestral human viruses circulated, with each introduction marked by decade and clade (Anderson et al., 2016, 2021).

Similarly, the N2 gene is classified according to the year of introduction of human seasonal strain into swine while the N1 classification system follows the same scheme of the H1 gene (Anderson et al., 2016).

The first whole-genome based classification proposed in Europe grouped swIAV into 23 genotypes (A-W) (Watson et al., 2015), subsequently updated in 2020 with the description of 11 additional genotypes (AA-AP) (Henritzi et al., 2020). In Italy, additional new swIAV genotypes were described between 2013 and 2017, and identified as: 1–4, 6–12, 26, 27, 29–32 (Chiapponi et al., 2022) and Novel2013 (Beato et al., 2016), not falling into the A-W and AA-AP classifications.

In the present study, we report the characterization of swIAV circulating in a densely populated pig area in Northeast Italy, through continuous monitoring in pig farms from 2013 to 2022. Such approach allowed the identification of regional and farm-based genetic clustering highlighting an unexpected genetic diversity depicted over time and multiple introductions within the same pig farm. Our data offers insights for monitoring swIAV genetic diversity.

## 2 Materials and methods

### 2.1 Study period and sampling strategy

Passive surveillance for swIAV was conducted in three North Eastern Italian regions over a nine-year period between 2013 and 2022. Samples included in the study originated from: Veneto (VEN), Friuli Venezia Giulia (FVG) and Trentino Alto Adige (TAA) regions. All samples, including nasal swabs, lungs, oral fluids, were submitted to the laboratory by field veterinarians following a clinical suspicion or a necropsy examination. Samples originated from different production type farms: farrow, fattening, weaning site and family farms (below 50 pigs).

Of note in 2013, a farm was monitored nearly every month for 6 months following an H1N1pdm outbreak, alongside the introduction of a targeted vaccination program against the H1N1pdm virus, authorized under derogation (Mughini-Gras et al., 2015).

Moreover, from January 2019 to December 2021, the launch of a 3-year research project funded by the Italian Ministry of Health enhanced the passive surveillance for swIAV, aiming to improve the monitoring of swIAV circulation by increasing the number of pig farms screened. Field veterinarians were incentivized to submit samples providing swIAV testing for free using the project budget and requesting a minimum of 15 swabs per pig farm.

### 2.2 swIAV detection and characterization

Nasal swabs and lung tissues (20–40 mg) were diluted in 1 mL of minimal essential medium (MEM) (Gibco, Waltham, Massachusetts, United States), 0.5% albumin and HEPES (Sigma Aldrich, St. Louis, Missouri, United States) buffer supplemented with 1% antibiotics (Pen/Strept, Euroclone, Italy). Lung tissues were homogenized at a 1:10 dilution (w/v) in 600 μL of PBS, using the TissueLyser II instrument (QIAGEN, Hilden, Germany). For each lung sample, a bronchial swab was also collected to increase swIAV detection rate. The oral fluids were analysed without any pre-treatment. Swabs were tested in pools of maximum five. The High Pure RNA Isolation Kit (Roche, Basel, Switzerland) was used for nasal and bronchial swabs and oral fluids, while the High Pure RNA Tissue Kit (Roche, Basel, Switzerland) was used for lung tissues. Alternatively, RNA extraction was carried out using the automated KingFisher Flex method (Thermofisher Scientific, Waltham, Massachusetts, United States) with the ID Gene Mag Universal Extraction Kit (IDvet Genetics, Grabels, France). To assess RNA extraction efficiency, a universal heterologous RNA control, Intype IC-RNA (Indical Bioscience GmbH, Leipzig, Germany), was added to each sample during the extraction phase at a 1:10 (v/v) ratio of the total elution volume. To screen samples a real-time reverse transcription polymerase chain reaction (rRT-PCR) protocol targeting the conserved M gene was applied (Hoffmann et al., 2006, 2010). Only samples and pools of swabs with an eligible cycle threshold (ct <30) were subtyped individually using another molecular method. Samples collected between 2013 and 2017 and testing positive for swIAV were HA and NA sub-typed using a multiplex RT-PCR assay (Chiapponi et al., 2012). Positive swIAV samples collected from 2018 were sub-typed by a validated multiplex real-time RT-PCR according to Henritzi et al. (2016) method. swIAV screening and sub-typing real time RT-PCRs were carried out using the AgPath-ID^™^ One-Step RT-PCR Reagents kit (Thermo Fisher Scientific, Waltham, Massachusetts, United States) and the CFX96 instrument (BIORAD, Hercules, California, United States). HA uncharacterized samples by real time RT-PCR (Henritzi et al., 2016) were tested in series, firstly by the multiplex RT-PCR assay (Chiapponi et al., 2012) and then by a “pan-HA” RT-PCR by Henritzi et al. (2016) using the one step RT PCR kit (QIAGEN, Hilden, Germany) and the SuperScript^™^ III one-step RT-PCR system with Platinum^™^ Taq DNA Polymerase (Invitrogen—Thermo Fisher Scientific, Waltham, Massachusetts, United States), respectively. Both assays were performed on the S1000 Thermal Cycler (BIORAD, Hercules, California, United States) or on ABI 9700 Thermal Cycler (Applied Biosystem, Waltham, Massachusetts, United States), respectively. The resulting amplified products were visualized either on a 7% acrylamide gel stained with silver or loaded in the LabChip GX Touch/GXII Touch instrument (PerkinElmer, Waltham, Massachusetts, United States).

### 2.3 Virus isolation

Virus isolation on cell cultures were carried out on Cancer coli-2 (Caco-2 ATCC^®^ HTB-37^™^) or Madin-Darby canine kidney-2 (MDCK ATCC^®^ CCL-34^™^) cell lines for samples showing ct values below 30. Positive swIAV samples were diluted 1:10 (v/v) in MEM (Gibco, Waltham, Massachusetts, United States) 0.5% Albumin and HEPES (Sigma Aldrich, St. Louis, Missouri, United States) supplemented with 1% antibiotics (Pen/Strep, Euroclone, Italy), and, after an incubation at +37°C for 2 h, 500 μL were used to infect a confluent cell monolayer. Three blind passages were performed (Chiapponi et al., 2010). The presence of swIAV related cytopathic effect (CPE) was confirmed by real time RT-PCR (Hoffmann et al., 2006, 2010) and swIAV isolates were HA and NA sub-typed (Henritzi et al., 2016) and subjected to whole genome sequencing as previously described. Virus isolates were deposited in the IZSVe Biobank (ID Biobank: IZSVe VIR SCT3 LVD 1–12; 42–89; 489–816) and in the European Virus Archive Global (EVAG).

### 2.4 Whole genome sequencing

RNA was extracted from a selection of positive swIAV isolates using the QIAamp Viral RNA Mini Kit (QIAGEN, Hilden, Germany), following the manufacturer’s instructions. All positive samples for swIAV presenting ct <30 were subjected to whole genome sequencing. The extracted RNA was then reverse-transcribed and amplified using the SuperScript^™^ III one-step RT-PCR system with Platinum^™^ Taq High Fidelity DNA Polymerase (Thermo Fisher Scientific, Waltham, MA, United States) and the primer pair MBTUni-12-DEG-new (5′-GCGTGATCAGCRAAAGCAGG-3′) and MBTUni-13 (5′-ACGCGTGATCAGTAGAAACAAGG-3′), which target conserved regions at the ends of the eight gene segments of IAV, as described in Fusaro et al. (2019). Sequencing libraries were prepared using either the Nextera XT DNA Sample preparation kit (Illumina, San Diego, CA, United States) or the Illumina DNA prep kit (Illumina, San Diego, CA, United States) and sequenced on a MiSeq instrument (Illumina, San Diego, CA, United States) using the 2 × 150 bp, 2 × 250 bp, 2 × 300 bp PairedEnd (PE) mode, based on the average insert size and the library preparation kit used.

### 2.5 Reference-based assembly of reads

The raw sequencing data was quality-filtered by removing reads that met the following criteria: (a) containing more than 10% undetermined bases (“N”), (b) having more than 100 bases with a quality (*Q*-score) below 7, and (c) being duplicates. For the remaining reads Illumina Nextera XT adapter trimming was performed using scythe v0.991^1^ adapters, as well as amplification primer trimming using trimmomatic v0.32. Additionally, low-quality ends of each read were trimmed using sickle v1.33.^2^ Reads shorter than 80 bases or unpaired after the previous filters were discarded.

To determine the most suitable reference sequence for creating the consensus sequence for each sample using reference-based assembly, 50,000 randomly selected high-quality reads from each dataset were compared against the non-redundant database of all known nucleotide sequences (NT, downloaded on February 12, 2018) using BLAST v2.7.1+. The most frequent hits corresponding to the complete eight segments of the swIAV were chosen to create the reference sequence. The high-quality reads were aligned against the previously identified reference sequence using BWA v0.7.12 with standard parameters. The alignment was processed with SAMtools v1.6 to convert it to the BAM format and sort it by position. Variants (SNPs and indels) were identified using LoFreq v2.1.2. Following the recommended workflow for LoFreq, the alignment was preprocessed with Picard-tools v2.1.0^3^ and GATK v3.5 to correct potential errors, realign reads around indels, and recalibrate base quality. Subsequently, LoFreq was used on the fixed alignment with the “--call-indels” option to produce a VCF file containing both SNPs and indels. From the final set of variants, indels with a frequency below 50% and SNPs with a frequency below 25% were discarded. To generate the consensus sequence, the reference sequence was appropriately modified according to the following rules: (a) at a given position j, if the sequence coverage was insufficient (<10X) to reliably identify a variant, an “N” base was added; (b) at a given position j, if the sequence coverage was sufficient (≥10X) to reliably identify a variant but no SNP was identified, the base present in the reference sequence at position j was added; (c) at a given position j, if the sequence coverage was sufficient (≥10X) to reliably identify a variant and at least one SNP was identified, the base was added using the IUPAC nucleotide code^4^ according to the present nucleotides. Consensus sequences generated for this study were uploaded in Genbank or GISAID (Supplementary Table 1).

### 2.6 swIAV genotyping

Each gene segment was assigned to one of the lineages defined by Watson et al. (2015). Representative samples from each lineage were included in every phylogenetic tree to facilitate classification of the sequences under study. Once all gene segments of each sample were classified, genotypes were determined based on samples genetic constellation, following the nomenclature used by Watson et al. (2015). To identify HA lineages and their corresponding subclades, we also followed the subclade classification described by Anderson et al. (2016), as implemented on the BV-BRC webserver.^5^

### 2.7 Phylogenetic analysis

The dataset of each segment was populated with sequences that had the highest similarity to the ones we obtained. These sequences were chosen from the NCBI public database using the BLAST algorithm (Altschul et al., 1990). Phylogenetic analysis was performed using the Maximum Likelihood method implemented in IQ-Tree v2.2.2.6 (Minh et al., 2020). The best substitution model was identified using the ModelFinder method of IQ-Tree, using BIC as the optimality criterion (Kalyaanamoorthy et al., 2017) and branches support values were calculated using the non-parametric bootstrap method (1,000 replicates). Phylogenetic trees were visualized with Figtree v1.4.3^6^ and figures were edited with Inkscape.^7^

### 2.8 Production of haemagglutinating swIAV antigens

Eight swIAV isolates, representatives of different gene clusters, were selected for the production of hemagglutinating antigens. swIAV isolates were concentrated using Amicon Ultra-15100k tubes (Merck Millipore, Burlington, Massachusetts, United States) to increase the HA titre that was tested before and after the concentration step according to WOAH manual (World Organisation for Animal Health, 2023). The virus concentration and infection steps of Caco-2 cells were repeated several times until viral stocks reached the HA titer of 1:512. Each produced batch was quality controlled by bacteriological examination. Virus inactivation was performed using β-propiolactone at 1:2,000 dilution (v/v) and the mixture incubated at +37°C in a water bath with agitation for 3 h. Complete virus inactivation was checked by virus isolation, as described previously.

### 2.9 Production of hyperimmune swine sera

Four-week-old swine of about 10 kg were used to produce four polyclonal antisera against four selected hemagglutinating antigens: one H1huN2 (A/swine/Italy/18-170856-1/2018), two H1avN2 (A/swine/Italy/13-163032/2013 and A/swine/Italy/19-163539-11/2019) and one H1huN2 (A/swine/Italy/13-139329/2013). Prior to the immunization, all the swine were serologically tested for swIAV and porcine reproductive and respiratory syndrome (PRRS) using ELISA commercial kits. The immunization protocol was based on previous experiments identified based on a publication regarding the antigenic analysis of American-origin swIAV (Lorusso et al., 2011) and consisted of three different immunizations 15 days apart injecting intramuscularly a 1:1 (v/v) antigen and adjuvant mixture in the thigh region (MONTANIDE ISA 201 VG, SEPPIC, France). Three weeks after the third immunization, blood was collected from each animal, serum separated and stored at −20°C until use. As quality controls, each serum sample was serologically tested for PRRS virus, Aujeszky’s disease, Porcine Circovirus Type 2, swine vesicular disease and classical swine fever by ELISA tests. All animal care and procedures adhered to the Guide for the Care and Use of Laboratory Animals and Directive 2010/63/EU for animal experiments (National law: D.Lgs. 26/2014), approved by the Italian Ministry of Health (Authorization Number: 248/2019-PR).

### 2.10 Cross HI tests

Hyperimmune swine sera were used to carry cross HI tests against the haemagglutinating swIAV antigens produced in this study. As controls in the HI test were also included four positive swIAV swine serum and four positive haemagglutinating antigens, corresponding to each subtype. Serum samples were tested individually. Each HI experiment was carried out in duplicate, and the geometric mean titre (GMT) calculated.

### 2.11 Incidence calculation

Incidence rates by month were calculated. The population at risk was defined as the number of herds tested in the month. Herds having a positive result during previous months were excluded from the population at risk. Cases were herds with a positive result in the month.

## 3 Results

### 3.1 Spatio-temporal distribution of swIAV in Northeast Italy

During the study period (2013–2022) and in the three Northeast Italian regions, VEN, FVG, and TAA, 253 farms were tested for swIAV, for a total of 471 sampling events. 144/471 (30.6%) batch of samples collected from 96/253 (37.9%) farms resulted positive (Table 1; Supplementary Table 2).

**Table 1:** Tested swine farms and samples per year.

Year	Tested samples	Positive samples	% positive samples	Farms	Positive farms	% positive farms
2013	227	31	13.7%	5	3	60.0%
2014	437	56	12.8%	25	5	20.0%
2015	67	15	22.4%	16	4	25.0%
2016	63	16	25.4%	21	5	23.8%
2017	478	28	5.9%	36	14	38.9%
2018	440	47	10.7%	50	10	20.0%
2019	359	101	28.1%	85	26	30.6%
2020	369	110	29.8%	91	28	30.8%
2021	405	104	25.7%	67	20	29.9%
2022	211	71	33.6%	75	29	38.7%
Total	3,056	579	18.9%	471	144	30.6%
Total (counted once)	—	—	—	253	96	37.9%

**Figure 1:**
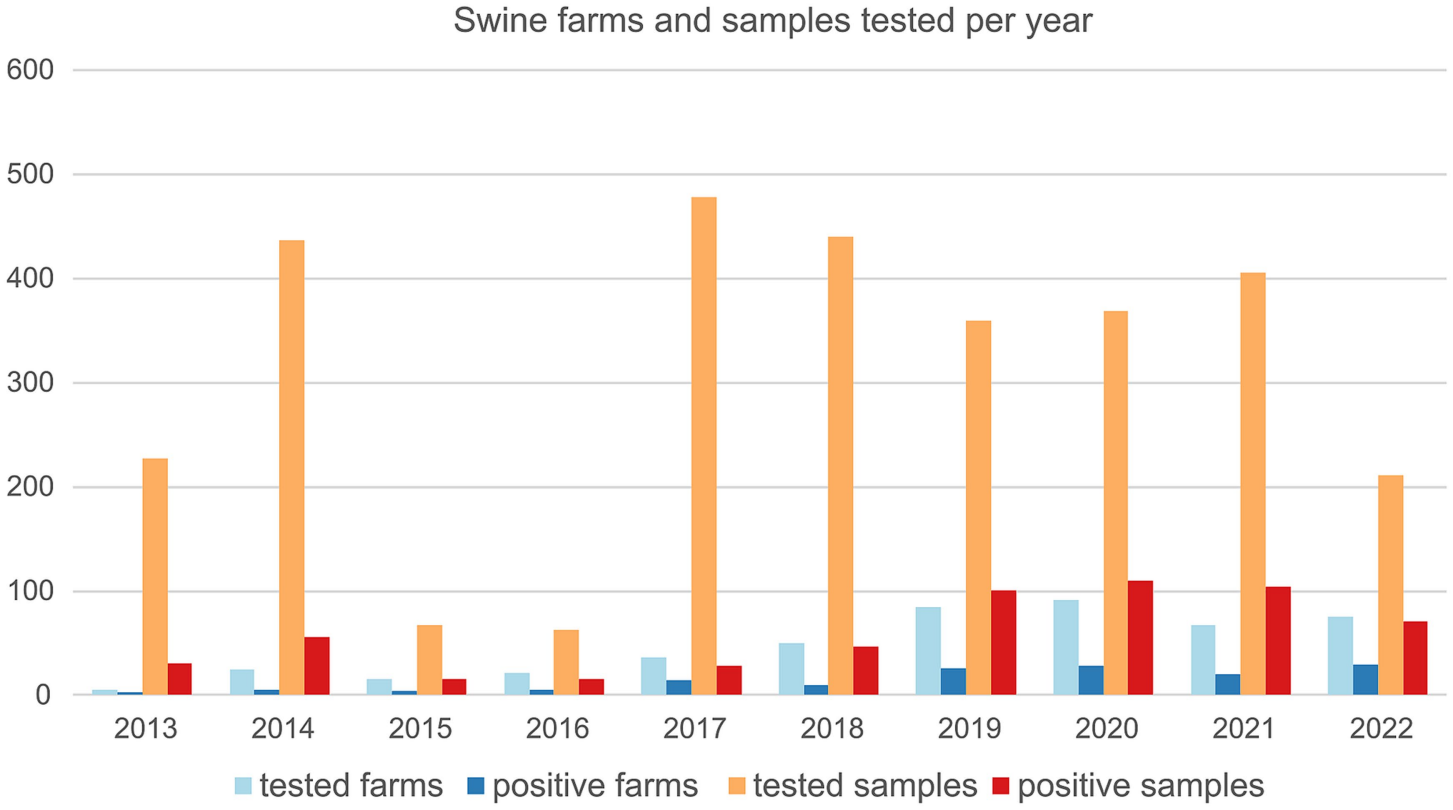
Number of swine farms and number of samples tested for swIAV by real time RT-PCR between 2013 and 2022 and relative positives.

**Figure 2:**
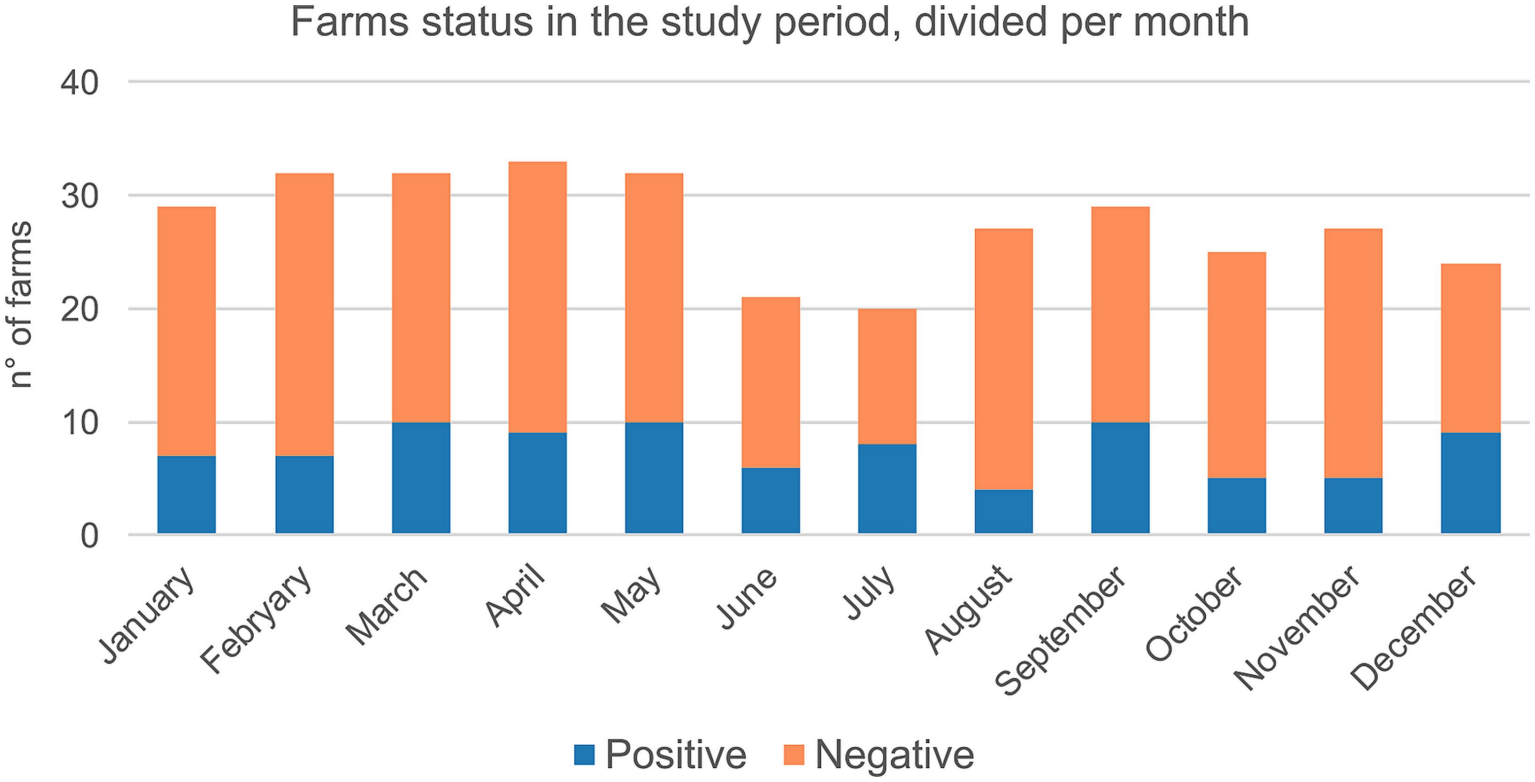
Cumulative number of farms tested for swIAV by real time PCR between 2013 and 2022 categorized per month and status (i.e., positive/negative).

Swine farms were distributed in the VEN region (*n* = 172) with 38.4% positive, FVG (*n* = 77) with 37.7% positive and TAA (*n* = 4) with 25% positive (Supplementary Table 2). The majority of tested farms were located in high densely populated swine provinces (i.e., Padova-PD; Verona-VR; Treviso-TV in Veneto region and Pordenone-PN and Udine-UD in FVG) where 34.4% (UD) to 55.1% (VR) resulted positive (Supplementary Table 2). The highest number of positive swine farms were between 2019 and 2022, particularly in 2022 (*n* = 29), followed by 2020 (*n* = 28), 2019 (*n* = 26) and 2021 (*n* = 20) (Figure 1). Based on the sampling conducted, the higher percentage of positive swine farms was detected in January, March, June, July, September, and December (Figure 2). Thirty-one (31.3%) percent of fattening farms, 46.8% farrows, 50% of weaning sites tested were swIAV positive. All (1.6%) the family farms tested were negative (Supplementary Table 4).

Total 3,056 samples were collected and tested for swIAV of which 579 positive (18.9%) (Table 1), distributed across the study period with the majority of positive samples identified in 2022 (33.6%) followed by 2020 and 2019 (29.8 and 28.1% respectively).

### 3.2 swIAV characterization by real time RT-PCR

Four hundred and twenty out of 579 positive swIAV samples (72.53%) were subjected to HA and NA subtyping (Table 2) of which more than 50% (227) were successfully subtyped as H1av (58.6%%), H1hu (13.7%), H1pdm (13.2%), H3 (3.5%)(Table 2), 25 samples (11%) resulted positive for two different subtypes.

**Table 2:** Subtype identified by real time RT-PCR and corresponding number of samples.

Subtype		Number of samples	
H1av	H1avN1	34	133
	H1avN2	99	
H1hu	H1huN1pdm	2	31
	H1huN2	29	
H1pdm	H1pdmN1	2	30
	H1pdmN1pdm	27	
	H1pdmN2	1	
H3	H3N1	3	8
	H3N2	5	
Coinfection	H1av + H1pdm N1 + N2	1	25
	H1av + H1huN1	20	
	H1av + H1huN2	2	
	H1avN1 + H3N2	2	
Partially untypable	H1avNX	2	147
	H1pdmNX	1	
	HXN1	28	
	HXN1pdm	8	
	HXN2	108	
Untypable		46	

### 3.3 swIAV genetic diversity and identification of two novel genotypes in Italy

**Figure 3:**
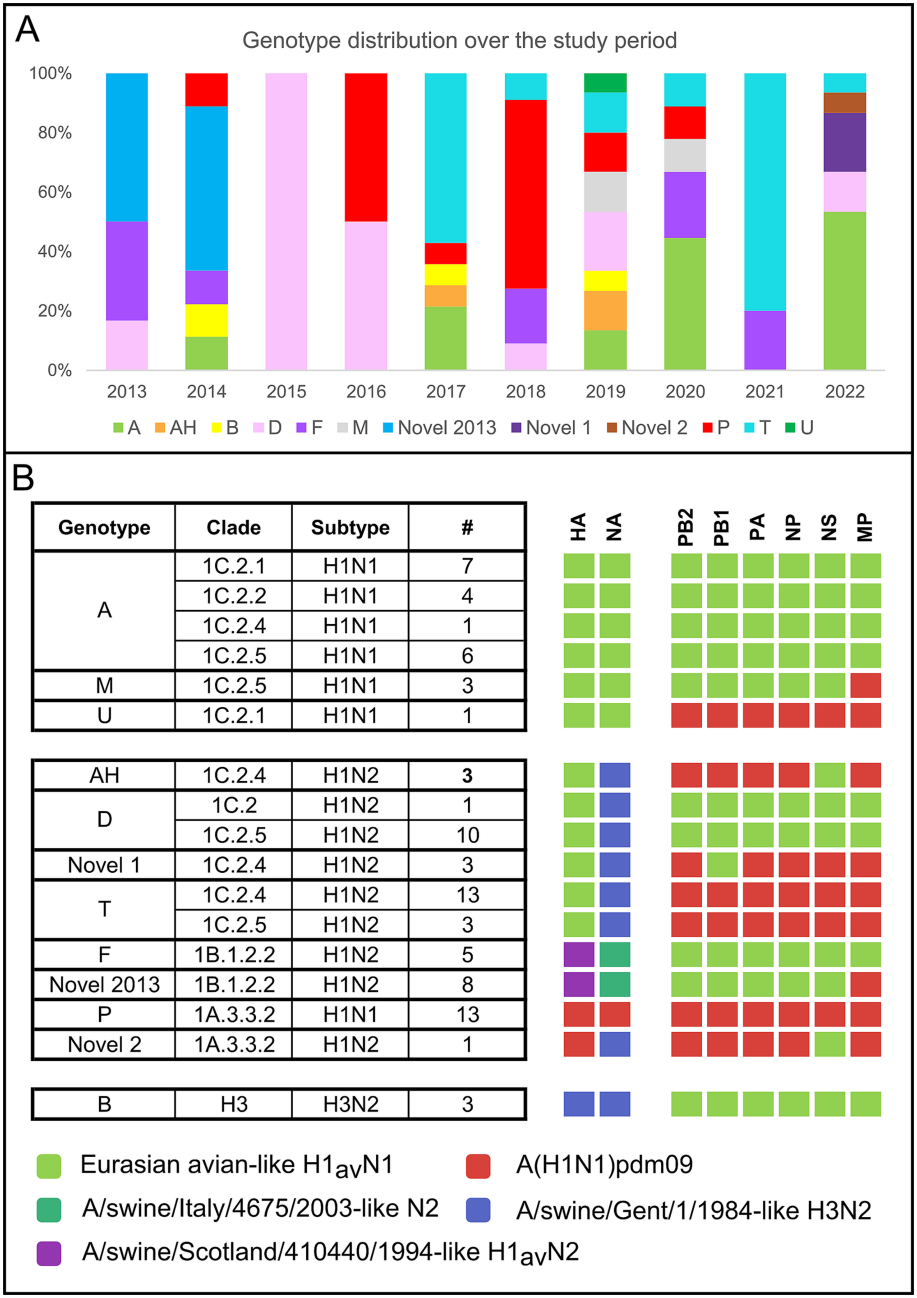
**(A)** Cumulative number and type of genotype and subtypes identified during the study period (2013–2022) according to the region of origin. **(B)** Cumulative number of genotypes identified during the study period divided per year.

Eighty-five samples were successfully sequenced (Supplementary Table 3). In addition, five influenza strains (A/swine/Italy/4159/2006, A/swine/Italy/711/2006, A/swine/Italy/4660-3/2009, A/swine/Italy/716/2006, A/Verona-Italy/2810/2009), used for cross HI tests were sequenced and included in the dataset. The sequenced samples originated from 45 swine farms located in two out of three regions under study (VEN and FVG) with the majority (67%) from the VEN region. The sequenced swIAV viruses were characterized as H1avN2 (*n* = 35); H1avN1 (*n* = 20), H1huN2 (*n* = 13), H1pdmN1 (*n* = 13), H1pdmN2 (*n* = 1) and H3N2 (*n* = 3) subtypes. The HA gene of the H1 viruses fell within lineage 1C (H1av, *n* = 55), 1B (H1hu, *n* = 13) and 1A (H1pdm, *n* = 14). All lineages were identified in the two regions with the exception of lineage 1B not detected in FVG. The lineage 1C viruses were the more heterogeneous with five subclades identified (1C.2 *n* = 1/85–1.2%; 1C2.1 *n* = 8/85–9.4%; 1C.2.2 *n* = 4/85–4.7%; 1C2.4 *n* = 20–22.3%; 1C.2.5 *n* = 22–25.9%) while lineage 1A viruses belonged to the subclade 1A3.3.2 (*n* = 14/85–16.5%) and lineage 1B to subclade 1B1.2.2 (*n* = 13–15.3%) (Figure 3). The NA genes of the avH1N2, pdmH1N2 and H3N2 subtype viruses all belong to the Gent/84 lineage clade, whereas the NA gene of the huH1N2 virus belongs to the A/swine/Italy/4675/2003 “human-like” N2 clade (Figure 3B). The H1avN2 subtype, belonging to 1C.2, 1C.2.4 and 1C.2.5 HA lineage, displayed the higher variability in genome composition with four distinct genotypes (D, T, AH and Novel 1), followed by the H1avN1 strains belonging to 1C.2.1, 1C.2.2 and 1C.2.5 HA lineage with three genotypes (A, M and U) (Figure 3).

**Figure 4:**
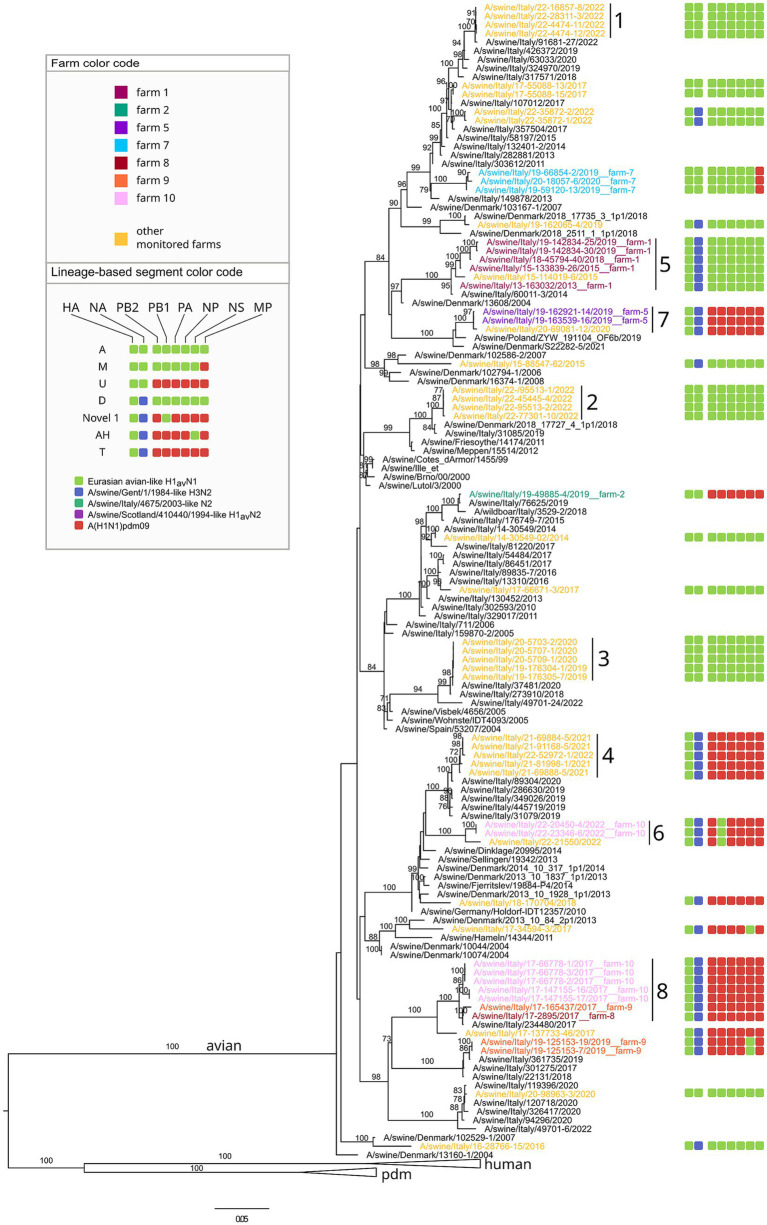
Phylogenetic tree of the HA segment of swIAV collected in North Italy between 2013 and 2022, viruses sequences were colored according to the farm of origin; the internal gene composition was provided beyond each sequenced strains. Phylogenetic tree of HA, with a focus on avian lineage. Only bootstrap values above 70 were shown. Cluster numbers were reported in black.

**Figure 5:**
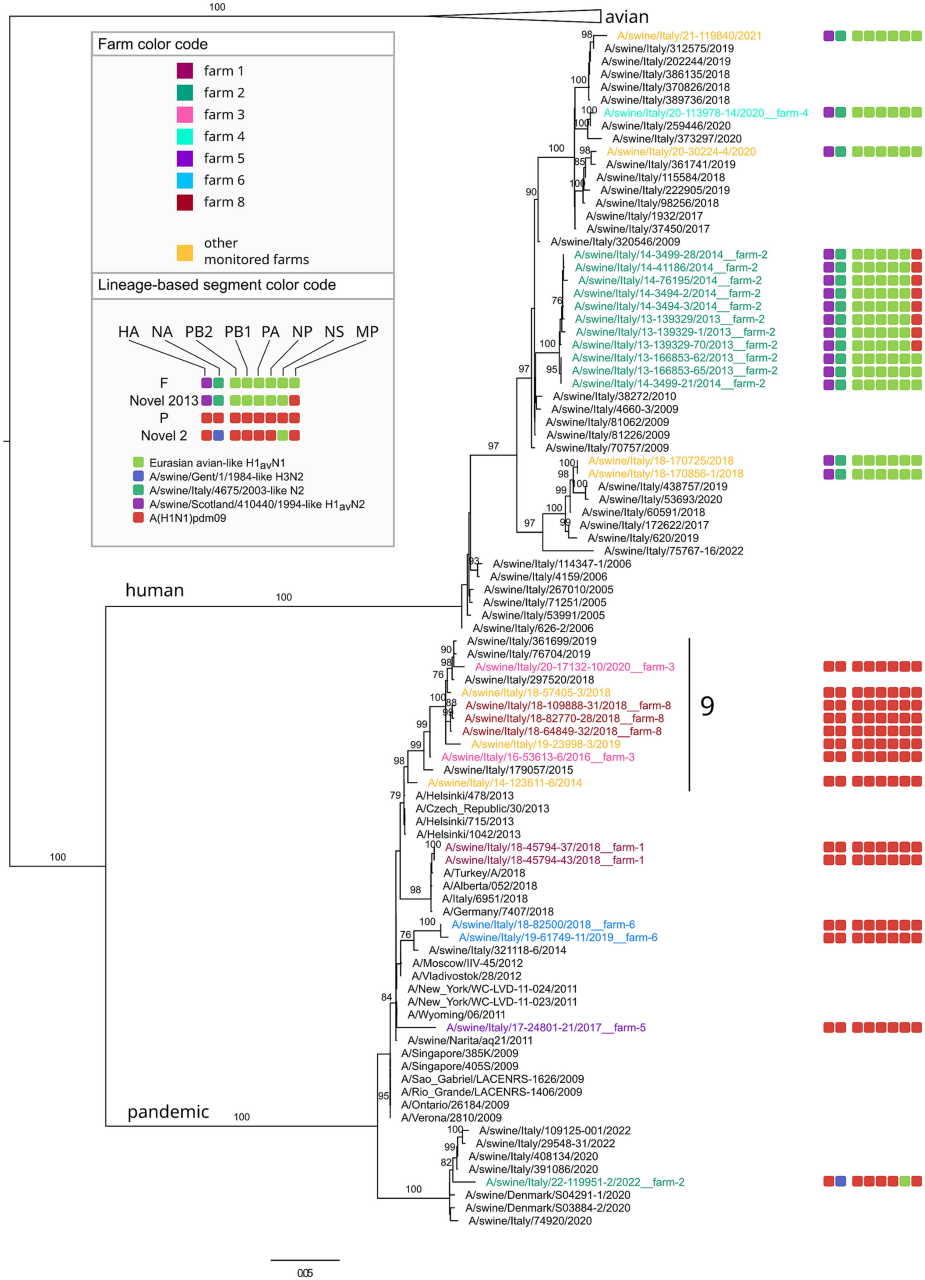
Phylogenetic tree of the HA segment of swIAV collected in North Italy between 2013 and 2022, viruses sequences were colored according to the farm of origin; the internal gene composition was provided beyond each sequenced strains. Phylogenetic tree of HA, with a focus on pandemic and human lineage. Only bootstrap values above 70 were shown. Cluster numbers were reported in black.

**Figure 6:**
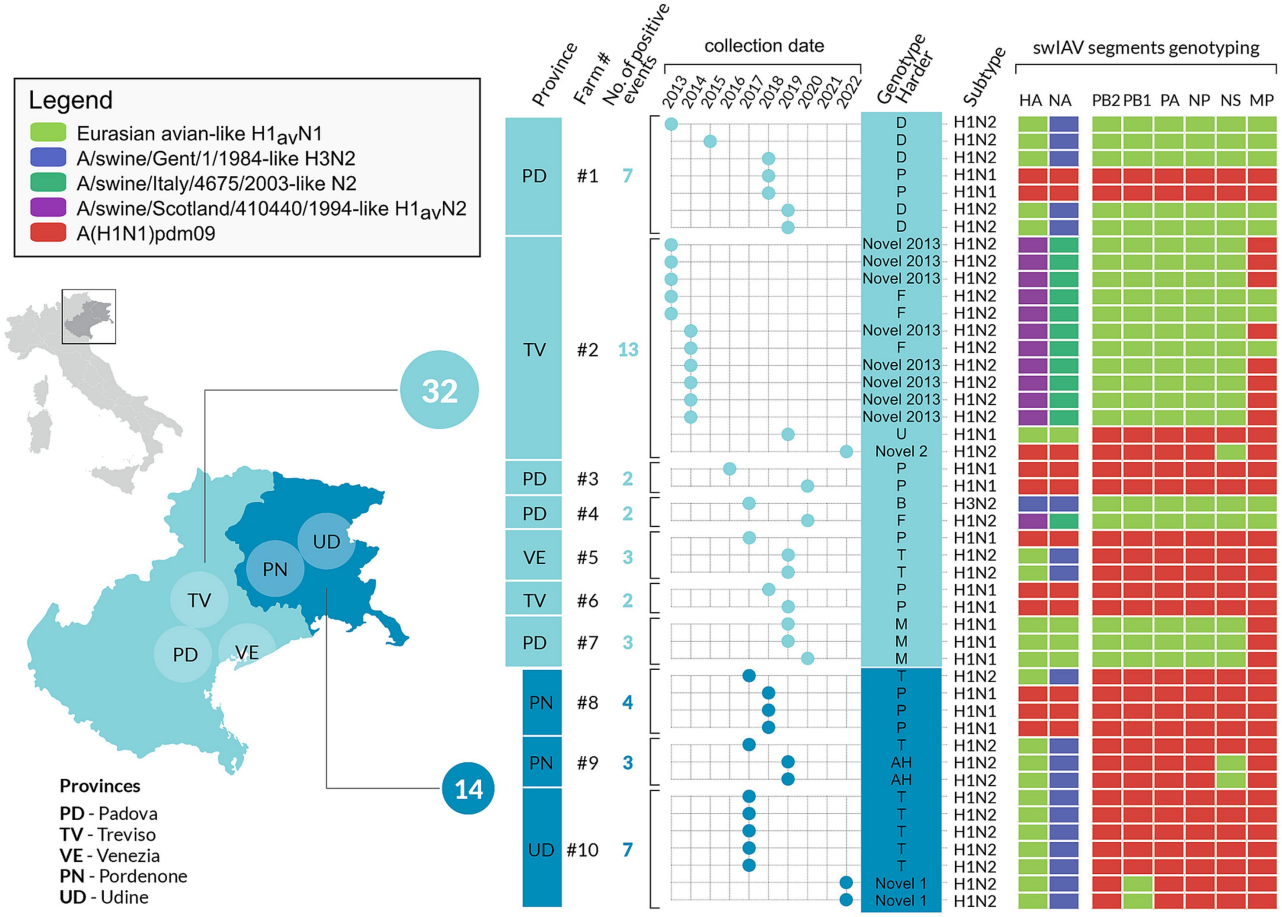
Infographics describing the 10 selected swine farms with repeated swIAV detection in the study area, between 2013 and 2022. The number and types of genotypes are represented per farm and year. The cumulative number of swIAV strains identified in each region is also provided.

Twelve distinct genotypes were identified during the study period of which 10 were already described in Italy and Europe (A, AH, B, D, F, M, P, T, U, Novel2013) (Figure 3A). Two genotypes both detected in 2022 but never described before in Europe, to the best of our knowledge, and not falling into any published available swIAV genetic classification, are here described for the first time as Novel 1 and Novel 2 (Figures 4–6 and Supplementary Table 3).

No intra-farm reassortment events were identified in the characterized swIAV strains included in the study.

The genotype A (*n* = 18, 21.2% of which 1C.2.1 = 8, 1C.2.5 = 5, 1C2.2 = 4, 1C.2.4 = 1) was the most frequently detected across the study period, followed by T (*n* = 16, 18.8% of which 1C.2.4 = 14, 1C.2.5 = 3), P (*n* = 13, 15.3%) and D (*n* = 11, 12.9%, of which 1C.2 = 1, 1C.2.5 = 10) (Figure 3B). The distribution of genotypes per year is presented in Figure 3A, showing the higher genotype variability in 2019 (eight genotypes) followed by 2014, 2017, 2020 and 2022 during which five distinct genotypes were detected (Figure 3A).

Regarding the genome constellation of the two novel genotypes identified in the study area and never described before in Europe, Novel 1 is an H1avN2 subtype carrying all the internal genes from the 1A lineage except for the PB1 being from 1C lineage (Figure 6 and Supplementary Table 3). Trees topology suggests that genotype Novel 1 could have been generated through a reassortment event in which the PB1 gene of the T genotype (pandemic origin) has been replaced with a PB1 of avian origin.

The Novel 1 genotype (A/swine/Italy/22-119951-2/2022) was detected in two distinct swine farms located in two FVG provinces and showed a high genetic identity, indicating a possible between-farm virus spread (Figure 4; Supplementary Figures 1–8). The Novel 2 is an H1pdmN2 subtype carrying all the internal genes from the P genotype except for the NS deriving from 1C lineage (Figure 5, Supplementary Table 3, and Supplementary Figures 1–8), was detected once in the VEN region in 2022 (A/swine/Italy/22-119951-2/2022).

Based on the HA phylogenetic tree, most of the H1 strains analysed in this study grouped together with other Italian viruses, with few exceptions (Figures 4, 5). In particular, some H1-1C strains (i.e., A/swine/Italy/15-88547-62/2015; A/swine/Italy/16-28766-15/2016, A/swine/Italy/19-162065-4/2019, A/swine/Italy/18-170704/2018, A/swine/Italy/17-34594-3/2017) cluster with strains from Denmark and some H1-1A strains (i.e., A/swine/Italy/18-45794-43/2018; A/swine/Italy/18-45794-37/2018; A/swine/Italy/17-24801-21/2017) cluster with human strains (Figure 5).

The phylogenetic trees topology showed a strong genetic clustering for all the eight genes of some of the analysed virus strains collected from different farms, suggesting a between-farm virus spread. Specifically, nine distinct genetic clusters were identified, each characterized by swIAV collected from at least two farms and conserved across the eight gene segments (Figures 4, 5; Supplementary Figures 1–8).

In detail, genetic cluster 1 was characterized by genotype A H1-1CN1 swIAVs identified in 2022 in three different swine farms from the same province, two of which located also in the same municipality and sharing the same owner (Figure 4). Cluster 2 was composed of four viruses belonging to genotype A H1avN1 swIAVs identified between April and July 2022 of which three were from the same province in VEN (reproduction farms) and the fourth in FVG (fattening farm). Cluster 3 was formed by six strains of the H1avN1 belonging to genotype A (*n* = 5), respectively and sharing the same internal genes, identified in the same province (Verona-VR) in neighboring municipalities between December 2019 and January 2020. Cluster 4 consisted of five H1avN2 swIAV strains belonging to genotype T identified in FVG (*n* = 3) and Veneto (*n* = 2) in May, June, July 2021 and July 2021 and April 2022, respectively. The three FVG strains originated from three different farms of the same supply chain, being reproduction, weaning and fattening farms, and two located in the same municipality.

Clusters 5 through 9 are described below, as they included viruses circulating on the same farm for more than 6 months.

### 3.4 Intra-farm swIAV circulation

Between 2013 and 2022, 10 swine farms presented at least two swIAV sequenced positive sampling events over a six-month period, of which seven (n. 1–7) located in the VEN region and the remaining three (n. 8–10) in FVG. Eight farms (n. 1–3, 5–8, 10) were reproduction farms and two were classified as finishing farms (n. 4 and 9). Forty-six swIAV were detected and sequenced across the 10 farms.

### 3.5 Farms in Veneto region

Three out of 10 farms, were positive only for one genotype (Farms 3, 6, 7) while all the other farms experienced the circulation of at least two different genotypes, with one farm (farm 2) hosting four different genotypes (Figure 6).

Farm 3 was subjected to the circulation of a single subtype H1pdmN1pdm (P genotype) identified in 2016 and 2020. According to the phylogeny, this two P genotypes clustered together with six other swIAV detected in FVG, three of which identified in the same farm (farm 8) in the same year, and the remaining three detected from three other farms in three different years (cluster 9 in Figure 5). Such clustering was observed for all genes except for the MP gene of one swIAV (A/swine/Italy/19-23998-3/2019). This cluster appeared related to human influenza strain (Figure 5), suggesting a likely human to pigs transmission event, followed by a persistence circulation and spread of this virus in the swine population in this geographic area.

Farms 6 and 7 experienced the circulation of a single genotype, P (H1pdmN1pdm) and M (H1avN1), respectively, detected during two and three sampling events over 2 years. The genetic clustering was indicative of a single introduction event in each farm.

Three farms (1, 4 and 5) were positive for two genotypes (Figure 6). In farm 1, P and D genotypes with the HA belonging to 1A.3.3.2 and 1C.2.5 lineages respectively, were detected in four distinct sampling events over 7 years (2013–2019). Genotype D was detected during four sampling events between 2013 and 2019, while P once only in 2018, co-circulating with the D genotype (Figures 5, 6). Strains belonging to genotype P were correlated, for all genes, to circulating human influenza strains detected in the same year in Italy, and other European and non-European countries (Figure 6). Genotype D viruses were all genetically closely correlated, constituting a distinct genetic cluster (n. 5), but showing an intra-farm virus evolution through the years.

Farm 4 was characterized by the presence of two different subtypes, H3N2 (in 2017) and H1huN2—(in 2020) corresponding to the B (H3N2) and F (1B.1.2.2) genotypes, suggesting two distinct introductions (Figure 6).

In farm 5, the P (1A.3.3.2) and T (1C.2.5) genotypes were detected in 2017 and 2019, respectively, interestingly, the P/2017 strain was likely introduced in the swine population by humans in the same year based on the phylogenetic analysis. Whether such an introduction occurred in farm 4 or in another farm remains unknown. Regarding the T genotype strains detected in 2019 they form a separate small conserved cluster (n. 7) for all genes, including 2020 strains from a farm of the same supply chain owned by the same farmer (Figure 6).

Farm 2 was characterized by the identification of four different genotypes over 10 years and namely: F (1B.1.2.2), U (1C.2.1), Novel2013 (1B.1.2.2) and Novel 2 (1A.3.3.2) (Figures 4–6). The F and Novel genotypes were detected in 2013 and 2014, while U and Novel 2 in 2019 and 2022, respectively. Based on the HA phylogenetic tree, it appears likely that three distinct introductions occurred in this farm as the 1B.1.2.2, 1C.2.1 and 1A.3.3.2 were detected in 2013–2014, 2019 and 2022, respectively (Figures 4–6). This is confirmed by the topology of the phylogenetic trees of the NA and PB2 genes (Supplementary Figures 1–3). The Novel2013 was described for the first time in this farm, experiencing the reassortment of PB1, PA, NP, NS and MP, generating the F genotype determined by the change of the MP gene (Figure 6; Supplementary Figure 7). The F and Novel2013 genotype strains circulating in farm 2 formed a distinct genetic group further divided in the three groups (Figures 5, 6) based on HA phylogeny.

### 3.6 Farms in Friuli Venezia Giulia region

Farms 8–10, were all genetically related. In farm 8, the T-1C.2.4 (in 2017) and P-1A.3.3.2 (in 2018) genotypes were detected (Figure 6), all clustering with strains circulating in farm 9 and 10 (Figures 4, 5). In particular, all the T strains genotype detected in 2017 in farms 8–10, formed a distinct genetic cluster (n. 8) for all genes (Figures 4, 5, Supplementary Table 3, and Supplementary Figures 1–9).

In addition, genotype P strains of farm 8 grouped with farm 3 strains forming a separate cluster (n. 9, described above) with other P strains detected in FVG from 2014 to 2019 (Figure 6).

Farm 9 experienced the circulation of two genotypes: T and AH (1C.2.4) in 2017 and 2019, respectively. This was the only farm where the AH genotype was identified.

Farm 10 underwent the circulation of two genotypes: T and Novel 1 (1C.2.4) in 2017 and 2022, respectively. Moreover, the Novel 1 strains grouped with another Novel 1 strain, the A/swine/Italy/22-21550/2022, isolated in a different farm of the same region forming a distinct small genetic cluster (n. 6) (Figures 4, 6).

### 3.7 swIAV antigenic characterization

A total of eight hyperimmune swine sera of which four produced in this study and four used as positive controls were used to carry out the cross HI tests against 12 antigens of which eight produced in this study and four used as positive controls (Table 3). The dataset consisted of a table of eight swine sera by 12 viruses resulting in 96 individual HI measurements. The antigenic analysis of circulating swIAV in Northeastern regions was evaluated by detecting antibodies against swine avian-like H1N1 (H1avN1), human-like H3N2 (H3huN2), human-like H1N2 (H1huN2), pandemic H1N1 (H1pdmN1pdm) viruses in swine serum samples using the hemagglutination inhibition technique. Minimum cross reactivity of anti-H1av sera with H1hu antigens was observed (<10 to 40) (Table 3). Anti H1hu sera showed similar low cross reactivity with H1av antigens with the exception for the A/swine/Italy/14-30549-02/2014 which showed higher cross-reactivity with all anti H1hu sera tested (640) (Table 3). In addition, minimum cross reactivity (10) was observed testing anti H1pdmN1 with H1av and H1hu antigens. Within the H1av antigens tested a decrease in cross reactivity was observed between 1C2.5 and 1C2.1 antigens as anti 1C2 sera showed less cross reactivity with 1C2.1 antigens (20) (Table 3). Regarding the H1pdm strain, the anti H1pdm serum against the 2009 strains decrease its reactivity when tested with a more recent antigens from swine detected in 2018 (from 320 to 80) (Table 3). The cross reactivity decreased according to the year of isolation of antigens used.

**Table 3:** Cross HI data using swIAV circulating in Northeastern regions of Italy.

Antigen			Serum							
			H1avN1	H1avN2		H1huN2			H1pdmN1pdm	H3N2
			1C2.1	1C.2.5	1C.2.5	\	1B.1.2.2	1B.1.2.2	1A.3.3.2	\
Subtype	HA lineage		*A/swine/Italy/711/2006*	A/swine/Italy/13-163032/2013	A/swine/Italy/19-163539-16/2019	*A/swine/Italy/4660-3/2009*	A/swine/Italy/13-139329/2013	A/swine/Italy/18-170856-1/2018	*A/Verona-Italy/2810/2009*	*A/swine/Italy/716/2006*
H1avN1	1C.2.1	*A/swine/Italy/711/2006*	640	160	320	<10	40	<10	<10	<10
	1C.2.1	A/swine/Italy/14-30549-02/2014	80	20	20	160	640	640	640	<10
	1C.2.5	A/swine/Italy/18-45794-40/2018	320	160	160	40	20	20	10	<10
H1avN2	1C.2.5	A/swine/Italy/13-163032/2013	160	**160**	160	40	10	10	10	<10
	1C.2.5	A/swine/Italy/19-163539-16/2019	40	160	**320**	20	20	80	10	<10
H1huN2	1B1.2.2	*A/swine/Italy/4159/2006*	<10	<10	<10	160	160	160	<10	<10
	1B1.2.2	A/swine/Italy/13-139329/2013	<10	<10	<10	320	**640**	640	<10	<10
	1B1.2.2	A/swine/Italy/18-170856-1/2018	40	20	20	1,280	1,280	**10,240**	<10	<10
	1B1.2.2	A/swine/Italy/18-170725/2018	<10	<10	<10	\	160	320	\	<10
H1pdmN1pdm	1A.3.3.2	*A/Verona/2810/2009*	<10	<10	<10	<10	320	160	320	<10
	1A.3.3.2	A/swine/Italy/18-109888-31/2018	80	10	10	<10	20	320	80	<10
H3N2	\	*A/swine/Italy/312583/2009*	<10	10	<10	<10	10	<10	<10	320

In italics antigens and antisera used as controls; in bold homologous antigen and antiserum, :\: data not available.

## 4 Discussion

Controlling swIAV is challenging due to the intricate nature of its epidemiology. While surveillance programs for swIAV have been established and implemented in some countries, active surveillance programs are lacking, and passive surveillance remains more widespread. The capacity for swIAV surveillance varies across countries, with limited resources hindering the surveillance efforts. Furthermore, the existing surveillance programs have not yielded adequate insights into the epidemiological features of swIAV as in many cases the genetic data generated is not accompanied with anamnestic and/or epidemiological data. The present study is the result of 9 years of passive surveillance for swIAV conducted in Northeastern regions of Italy with two combined approaches. On one side, a truly passive surveillance activity based on clinical suspicions from field veterinarians, on the other side an enhanced passive surveillance based on an awareness campaign conducted from the beginning of 2019 until the end of 2022, funded by the Italian Ministry of Health. Field veterinarians were requested to submit at least 15 nasal swabs per farm and swIAV testing was supported by the project. During the years of the awareness campaign (2019–2022) the tested farms and the number of samples increased over time, along with the percentage of positive farms detected suggesting the strategy implemented had a positive impact on swIAV surveillance. All swIAV positive farms were detected following observation of clinical signs indicating that swIAV characterized might be linked to worsen clinical conditions in animals. However, such observation was not coupled with the collection of anamnestic data, representing a limitation of this study, in order to provide robust hypotheses and grounds for further studies. No seasonality of swIAV detection was observed during the study period, confirming previous studies and field evidences (Diaz et al., 2017; Loeffen et al., 2009; Ma, 2020; Rose et al., 2013; Ryt-Hansen et al., 2019a, 2019b; Simon-Grifé et al., 2012) and suggesting that a constant swIAV monitoring through the year should be implemented. With reference to farm typology and animal category, this study showed that swIAV circulation was primarily detected in farrow and weaning animals (Diaz et al., 2017; Nelson et al., 2011; Ryt-Hansen et al., 2019a, 2019b).

Regarding the genetic heterogeneity of swIAV, all H1 subtypes and lineages were detected during the study period in Italy: with the H1avN2 frequently detected followed by the H1avN1. The H3N2 subtype was detected less frequently. Twelve different genotypes were characterized in 9 years in a relatively small area of Italy: the Northeast. Ten genotypes were previously described in Europe and/or Italy (Beato et al., 2016; Chiapponi et al., 2022; Henritzi et al., 2020) and two were newly identified in the present study. Between 2017 and 2020, in Italy, 24 different genotypes were detected most of which belonging to 1C lineage, with the most prevalent genotype being the F (1B.1.2.2-itN2), accounting for 38.6% of the circulating strains (Chiapponi et al., 2022). By contrast, in our study, the most prevalent was genotype A, with genotype F detected in a limited number of farms and in a limited period, indicating a regional based genetic clustering of swIAV in Italy. The type and number of swIAV genotypes identified per year during the study period appeared to be positively correlated to the number of tested farms. The highest number of genotypes was detected in 2019, one the year with the highest number of tested farm (*n* = 85). In recent years, swIAVs circulating in Italian pig farms showed significant genetic diversification, particularly among the most frequently detected subtypes H1N1 and H1N2 (Chiapponi et al., 2022), confirmed by this study. The H1N2 subtype circulating in Italy was the most variable, belonging to eight distinct H1 lineages (1C.2, 1C.2.1, 1C.2.2, 1C.2.4, 1C.2.5, 1B.1.2.1, 1B.1.2.2, 1A.3.3.2) in combination with three N2 lineages (it-N2, N2g, N2s) and pandemic- or avian-origin internal genes. Our study confirmed this data with the exception of subclade 1B.1.2.1 that was not detected. The 1C lineage was the most widespread, as detected in all the study area, and displayed a high degree of internal gene variability. During the study period, there was an increasing presence of the 1A strains in particular in 2018. The detection of H3N2 strains was infrequent, maintaining a stable genetic pattern, in line with previous studies (Chiapponi et al., 2022).

Nine distinct genetic clusters were identified, each composed of samples collected at different sampling events from the same farm or from different farms. For some clusters (i.e., 1, 3) the link among the farms of origin was evident sharing the same owner or being located in neighboring farms in high densely populated pig area such as the Verona province, for others the link could be hypothesized, but not confirmed due to the absence of anamnestic data. This highlights the need to couple the genetic characterization of circulating swIAV with a proper collection of anamnestic data such as: farm typology and management, origin and movement of animals and humans.

A noteworthy result is the identification of 10 farms in which swIAV were identified twice at least 6 months apart, highlighting a potential sustained/persistent circulation or multiple introductions. Human to animal transmission (farms 3 and 5) and an intra-farm virus evolution (farms 1, 6, 7) through the years (2013–2019), was evident in some farms. The intra-farm evolution observed indicated a continuous circulation, suggesting the implication of the type of swine farm organization and management. In addition, some farms experienced distinct swIAV introductions (farm 4) and other farms experienced the swIAV circulation clustering with swIAV in other farms (farms 3, 5, 8). Some strains widely circulated among farms, and this occurred for the T genotype in FVG region only, suggesting a regional based distribution of swIAV genotypes. Our analysis showed the presence of similar strains among farms located in different regions and this was the case of the P genotype, indicating a potential common use of service and/or movements of animals and personnel. No intra or inter-farm reassortment was observed in this study.

The farm organization and management system might be a factor influencing the circulation and distribution of genotypes and genetic clusters driven most probably by the human factor. In particular, the surveyed swine farms showed the introduction of different genotypes at a higher rate than the expected reassortment events suggesting that new introductions might be explained with the production cycle-based pig farming system, which ensures separation between production phases hampering reassortment events and/or the short life of pigs in the farming system. The biosecurity measures applied on swine farms may prevent the introduction and/or new introductions of swIAV and prevent a prolonged circulation of multiple strains, recognized as a crucial risk factor for reassortment events. The simultaneous circulation of different lineages and genotypes detected in the study area, however, poses risks for potential future reassortment events.

One genotype, here reported, was previously described in the same study area, i.e., Novel, herein defined as Novel2013 (Beato et al., 2016), subsequently described in a neighboring region between 2017 and 2020 as genotype 27 using a local nomenclature (Chiapponi et al., 2022). Two additional genotypes have been provisionally named Novel 1 and Novel 2, as they have never been described before in Europe, to the best of our knowledge. This highlights that the absence of a harmonized genotype nomenclature to appoint swIAV genotypes in Europe might generate difficulties in tracking correctly the novel genotypes and hampers a straightforward comparison of published data. Therefore, there is an urgent need for a unique nomenclature for genotypes in Europe and a more detailed submission of information for each publicly available deposited swIAV sequenced strains.

Regarding the attempt to investigate the antigenic relationship among the circulating swIAV strains circulating in Northeast Italy, data indicated a low cross reactivity among H1av, H1hu and H1pdm. In particular, a diverse cross-reactivity was observed among 1C lineage antigens, indicating potential antigenic differentiation among the 1C strains that must be further investigated. In addition, regarding the 1A lineage viruses tested, based on the dataset generated, the cross reactivity among the original 2009 strains and the swIAV from 2018 decreased, probably ascribed to temporal antigenic modification and/or adaptation to swine. The limitation of the antigenic characterization carried out is due to the restricted number of strains included. Although such studies are useful to track escape mutants and broadly cross reactive viruses for vaccine candidates selection, production of haemagglutinating antigens, with acceptable HA titres, and pig sera remain a major bottleneck. This may affect the timely generation of data of swIAV antigenic modifications that may have impacts on vaccine manufacturing and surveillance. However, this study led to the development of new antigens and hyperimmune sera useful for future antigenic characterization of Italian and European swIAV.

Continuous monitoring through the years in the same area, as described in the present study, allowed the tracking of swIAV circulation in the area and in pig farms and their evolution, shedding light over potential links among farms and therefore risk factors. In particular, our study provided evidence of the regional and farm based clustering of swIAV collected in Northeast Italy that might be used to better target swIAV surveillance for future monitoring. In addition, our study highlighted the importance of fully characterized the swIAV genome of positive farms and in conjunction to collect anamnestic data to better investigate drivers of swIAV introduction and circulation in the study area.

## Data Availability

The datasets presented in this study can be found in online repositories. The names of the repository/repositories and accession number(s) can be found in the article/Supplementary material.

## Footnotes

^1^ https://github.com/vsbuffalo/scythe

^2^ https://github.com/najoshi/sickle

^3^ http://picard.sourceforge.net

^4^ http://www.bioinformatics.org/sms/iupac.html

^5^ https://www.bv-brc.org/app/SubspeciesClassification

^6^ http://tree.bio.ed.ac.uk/

^7^ http://www.inkscape.org/

## Supplementary material

SUPPLEMENTARY FIGURE 1 Phylogenetic tree of the N2 segment of swIAV collected in Northeast Italy between 2013 and 2022. Only bootstrap values above 70 were shown.

SUPPLEMENTARY FIGURE 2 Phylogenetic tree of the N1 segment of swIAV collected in Northeast Italy between 2013 and 2022. Only bootstrap values above 70 were shown.

SUPPLEMENTARY FIGURE 3 Phylogenetic tree of the PB2 segment of swIAV collected in Northeast Italy between 2013 and 2022. Only bootstrap values above 70 were shown.

SUPPLEMENTARY FIGURE 4 Phylogenetic tree of the PB1 segment of swIAV collected in Northeast Italy between 2013 and 2022. Only bootstrap values above 70 were shown.

SUPPLEMENTARY FIGURE 5 Phylogenetic tree of the PA segment of swIAV collected in Northeast Italy between 2013 and 2022. Only bootstrap values above 70 were shown.

SUPPLEMENTARY FIGURE 6 Phylogenetic tree of the NP segment of swIAV collected in Northeast Italy between 2013 and 2022. Only bootstrap values above 70 were shown.

SUPPLEMENTARY FIGURE 7 Phylogenetic tree of the MP segment of swIAV collected in Northeast Italy between 2013 and 2022. Only bootstrap values above 70 were shown.

SUPPLEMENTARY FIGURE 8 Phylogenetic tree of the NS segment of swIAV collected in Northeast Italy between 2013 and 2022. Only bootstrap values above 70 were shown.

SUPPLEMENTARY FIGURE 9 Phylogenetic tree of the H3 segment of swIAV collected in Northeast Italy between 2013 and 2022. Only bootstrap values above 70 were shown.
