# Supplementary figures and images for: Swine influenza surveillance in Italy uncovers regional and farm-based genetic clustering
Source: Front Microbiol. 2025 Jul 21;16:1607204. doi: 10.3389/fmicb.2025.1607204 (PMC12318940; doi:10.3389/fmicb.2025.1607204)

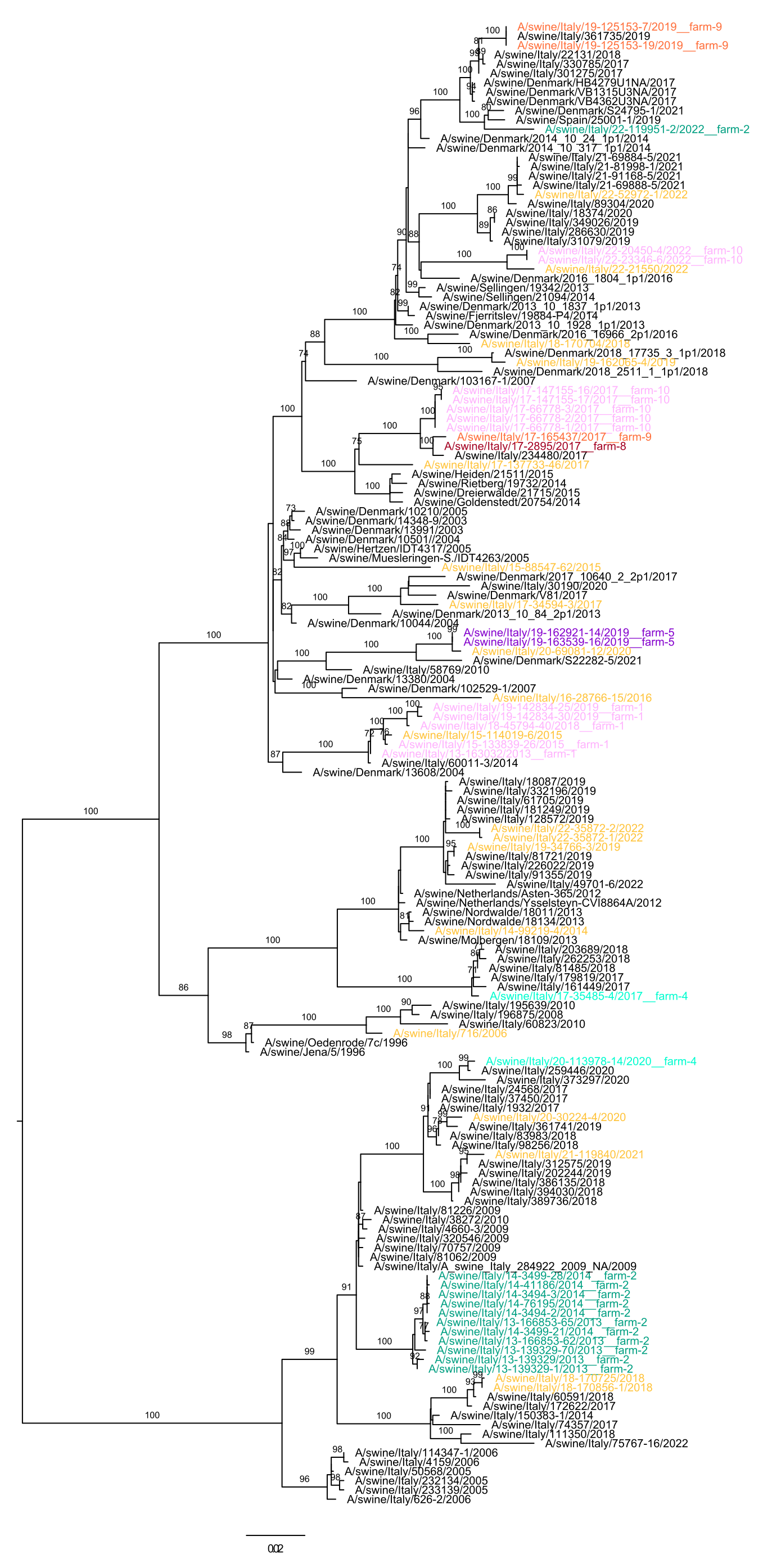

Supplement: SUPPLEMENTARY FIGURE 1 — Phylogenetic tree of the N2 segment of swIAV collected in Northeast Italy between 2013 and 2022. Only bootstrap values above 70 were shown. [file Image_1.tiff]

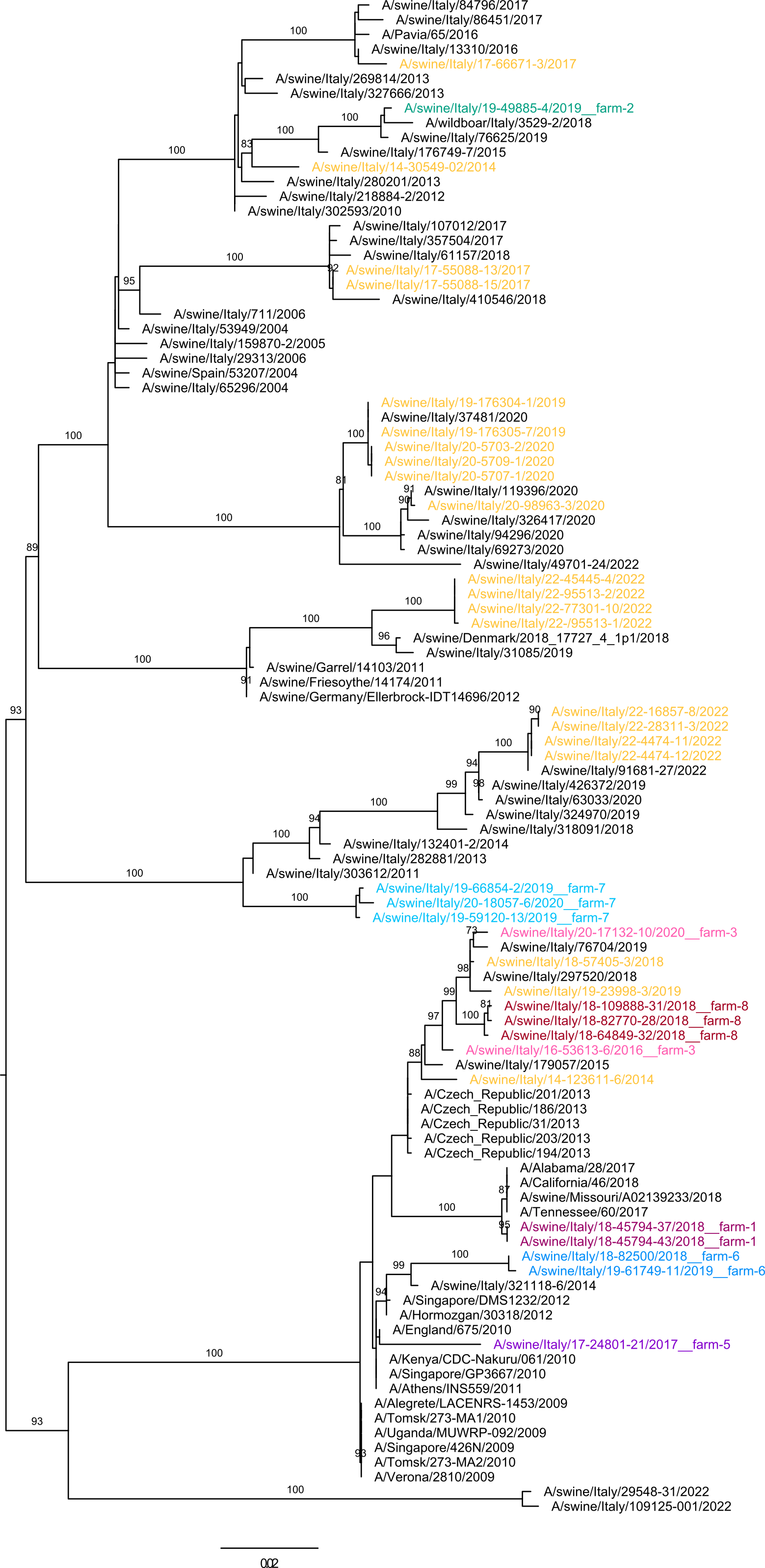

Supplement: SUPPLEMENTARY FIGURE 2 — Phylogenetic tree of the N1 segment of swIAV collected in Northeast Italy between 2013 and 2022. Only bootstrap values above 70 were shown. [file Image_2.tiff]

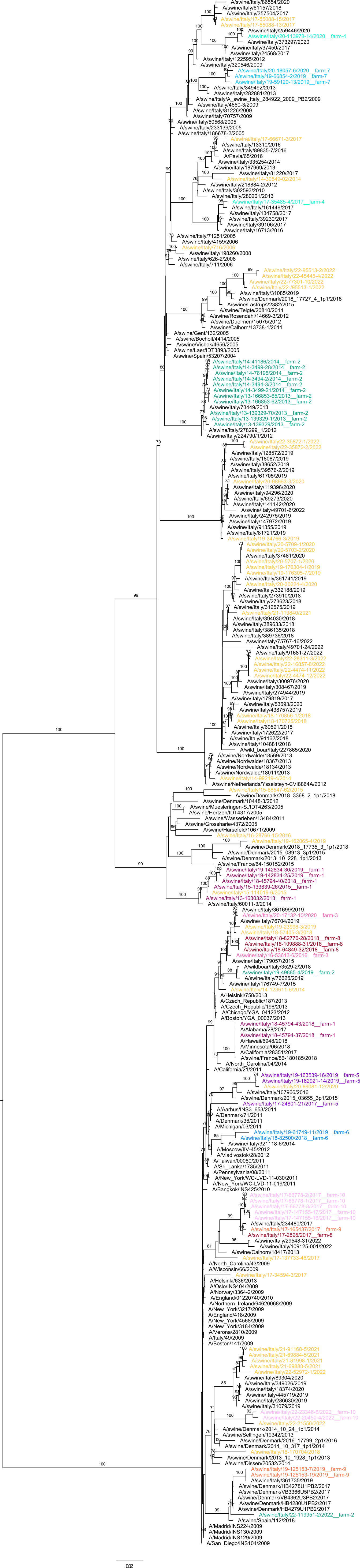

Supplement: SUPPLEMENTARY FIGURE 3 — Phylogenetic tree of the PB2 segment of swIAV collected in Northeast Italy between 2013 and 2022. Only bootstrap values above 70 were shown. [file Image_3.tiff]

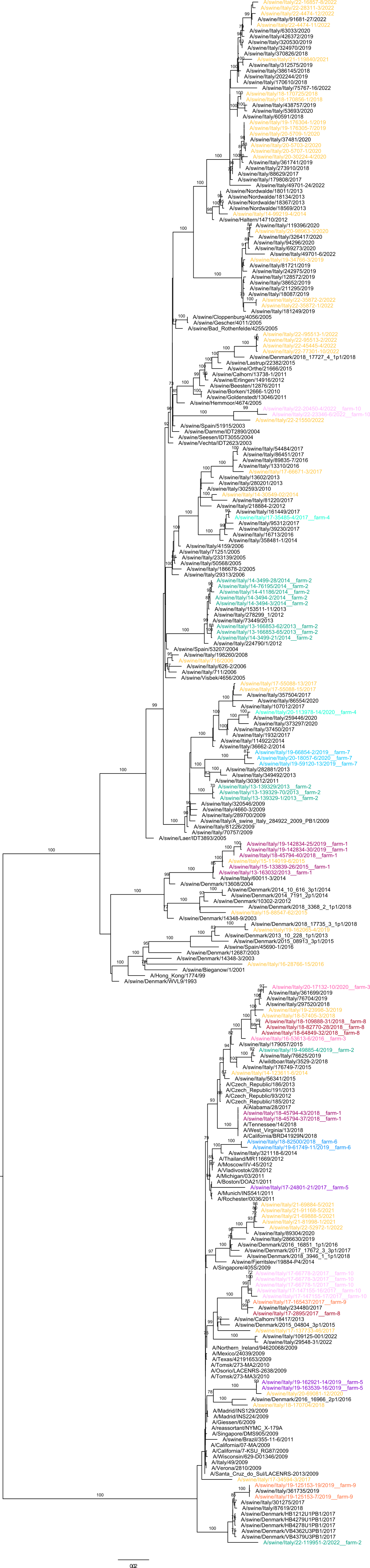

Supplement: SUPPLEMENTARY FIGURE 4 — Phylogenetic tree of the PB1 segment of swIAV collected in Northeast Italy between 2013 and 2022. Only bootstrap values above 70 were shown. [file Image_4.tiff]

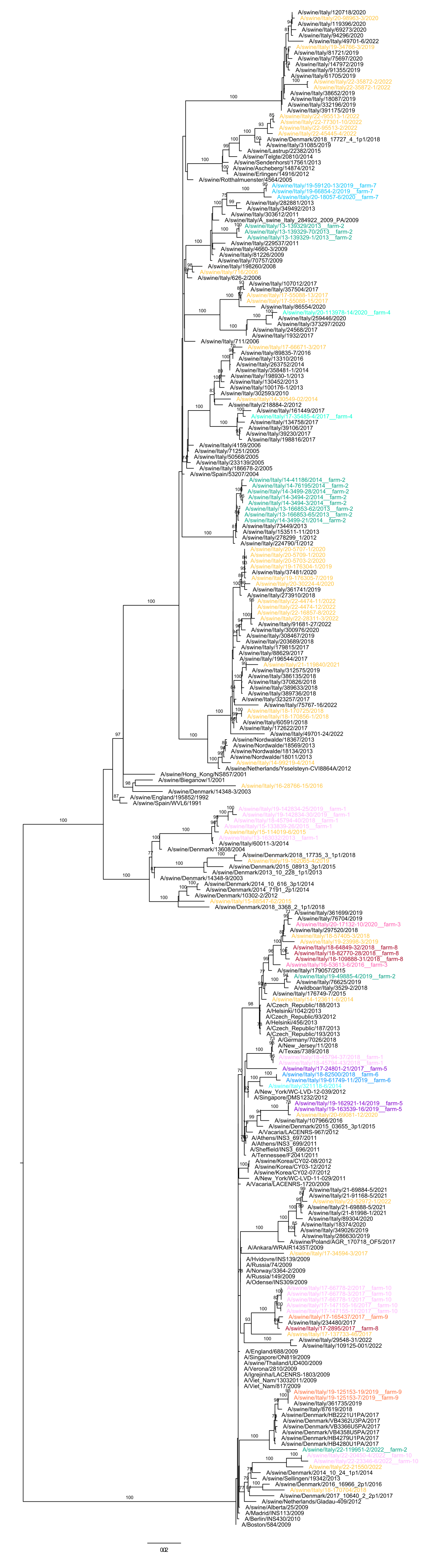

Supplement: SUPPLEMENTARY FIGURE 5 — Phylogenetic tree of the PA segment of swIAV collected in Northeast Italy between 2013 and 2022. Only bootstrap values above 70 were shown. [file Image_5.tiff]

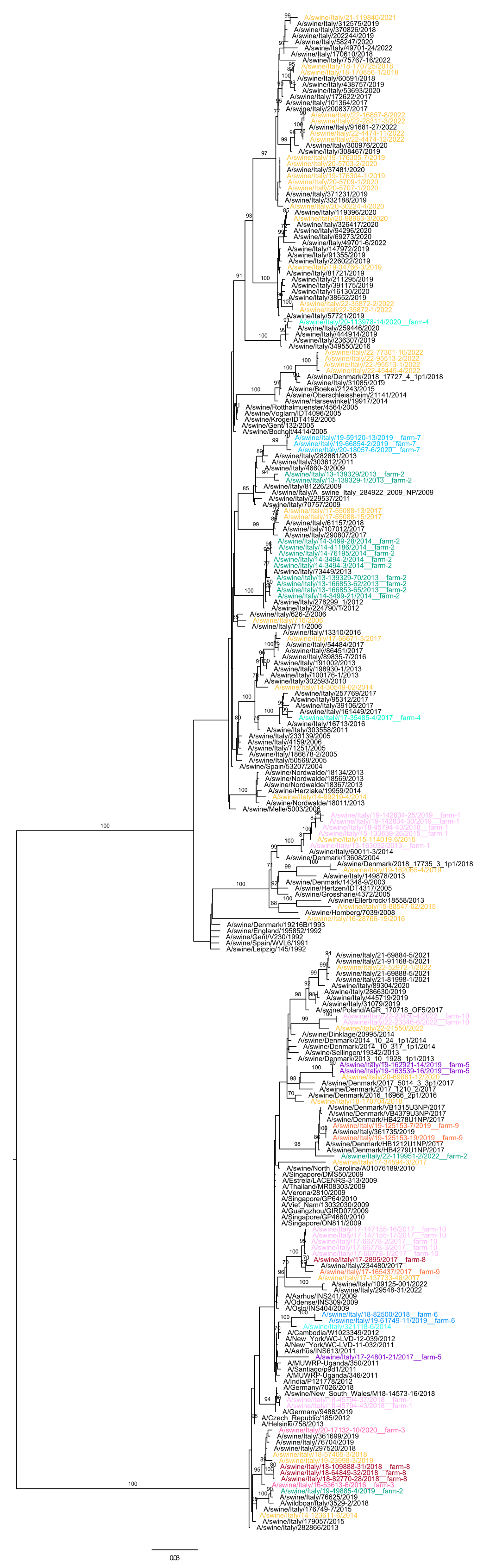

Supplement: SUPPLEMENTARY FIGURE 6 — Phylogenetic tree of the NP segment of swIAV collected in Northeast Italy between 2013 and 2022. Only bootstrap values above 70 were shown. [file Image_6.tiff]

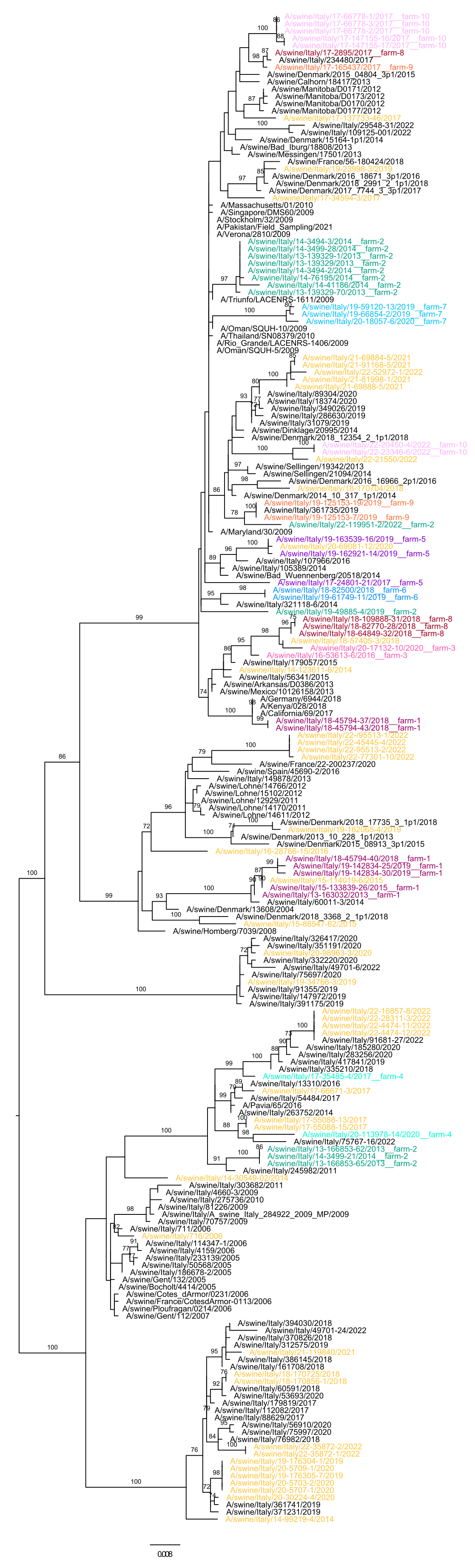

Supplement: SUPPLEMENTARY FIGURE 7 — Phylogenetic tree of the MP segment of swIAV collected in Northeast Italy between 2013 and 2022. Only bootstrap values above 70 were shown. [file Image_7.tiff]

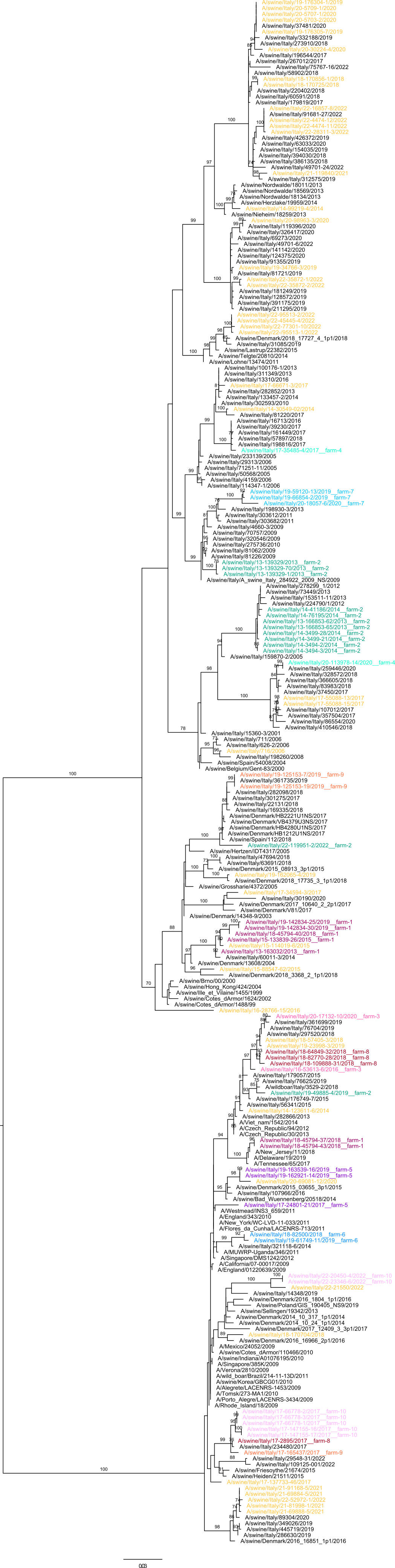

Supplement: SUPPLEMENTARY FIGURE 8 — Phylogenetic tree of the NS segment of swIAV collected in Northeast Italy between 2013 and 2022. Only bootstrap values above 70 were shown. [file Image_8.tiff]

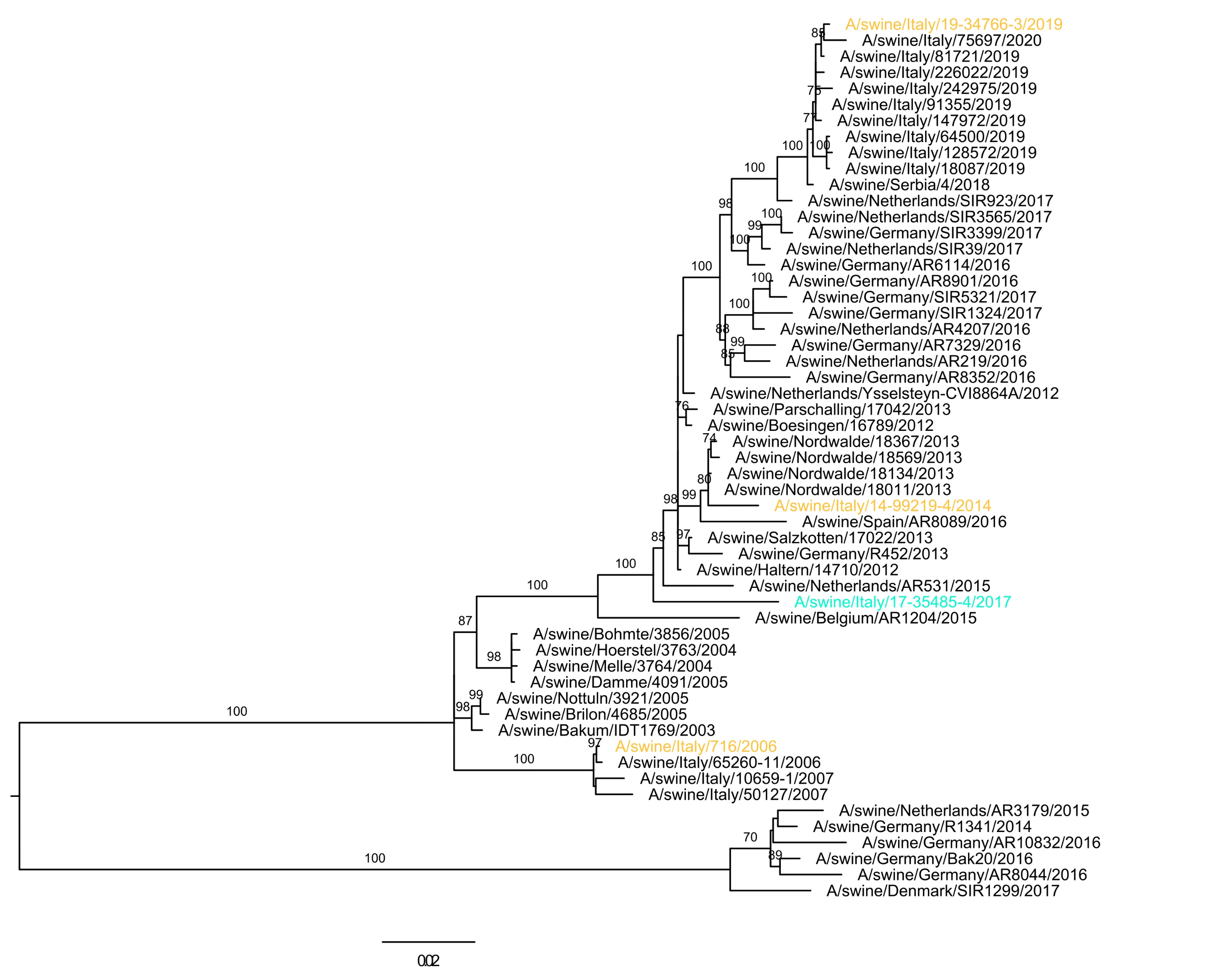

Supplement: SUPPLEMENTARY FIGURE 9 — Phylogenetic tree of the H3 segment of swIAV collected in Northeast Italy between 2013 and 2022. Only bootstrap values above 70 were shown. [file Image_9.tiff]
