# Supplementary material for: Swine influenza surveillance in Italy uncovers regional and farm-based genetic clustering
Source: Front Microbiol. 2025 Jul 21;16:1607204. doi: 10.3389/fmicb.2025.1607204 (PMC12318940; doi:10.3389/fmicb.2025.1607204)
Supplement: Supplementary file 10 [file Data_Sheet_1.pdf]

SUPPLEMENTARY MATERIAL INCLUDING TABLES AND FIGURES

Supplementary Table 1. List of strains with accession numbers included in the study.

Sequence name	PB2	PB1	PA	HA	NP	NA	MP	NS
A/swine/Italy/4159/2006	EPI3394283	EPI3394284	EPI3394282	EPI3394286	EPI3394279	EPI3394285	EPI3394281	EPI3394280
A/swine/Italy/13-166853-62/2013	EPI3867327	EPI3867328	EPI3867326	EPI3867330	EPI3867323	EPI3867329	EPI3867325	EPI3867324
A/swine/Italy/13-166853-65/2013	EPI3867335	EPI3867336	EPI3867334	EPI3867338	EPI3867331	EPI3867337	EPI3867333	EPI3867332
A/swine/Italy/14-3494-2/2014	EPI3867343	EPI3867344	EPI3867342	EPI3867346	EPI3867339	EPI3867345	EPI3867341	EPI3867340
A/swine/Italy/14-3494-3/2014	EPI3867351	EPI3867352	EPI3867350	EPI3867354	EPI3867347	EPI3867353	EPI3867349	EPI3867348
A/swine/Italy/14-3499-21/2014	EPI3867359	EPI3867360	EPI3867358	EPI3867362	EPI3867355	EPI3867361	EPI3867357	EPI3867356
A/swine/Italy/14-3499-28/2014	EPI3867367	EPI3867368	EPI3867366	EPI3867370	EPI3867363	EPI3867369	EPI3867365	EPI3867364
A/swine/Italy/22-119951-2/2022	EPI3867375	EPI3867376	EPI3867374	EPI3867378	EPI3867371	EPI3867377	EPI3867373	EPI3867372
A/swine/Italy/17-35485-4/2017	EPI3867383	EPI3867384	EPI3867382	EPI3867386	EPI3867379	EPI3867385	EPI3867381	EPI3867380
A/swine/Italy/19-59120-13/2019	EPI3867391	EPI3867392	EPI3867390	EPI3867394	EPI3867387	EPI3867393	EPI3867389	EPI3867388
A/swine/Italy/21-91168-5/2021	EPI3867399	EPI3867400	EPI3867398	EPI3867402	EPI3867395	EPI3867401	EPI3867397	EPI3867396
A/swine/Italy22-35872-1/2022	EPI3867407	EPI3867408	EPI3867406	EPI3867410	EPI3867403	EPI3867409	EPI3867405	EPI3867404
A/swine/Italy/22-35872-2/2022	EPI3867415	EPI3867416	EPI3867414	EPI3867418	EPI3867411	EPI3867417	EPI3867413	EPI3867412
A/swine/Italy/22-52972-1/2022	EPI3867423	EPI3867424	EPI3867422	EPI3867426	EPI3867419	EPI3867425	EPI3867421	EPI3867420
A/swine/Italy/22-28311-3/2022	EPI3867431	EPI3867432	EPI3867430	EPI3867434	EPI3867427	EPI3867433	EPI3867429	EPI3867428
A/swine/Italy/22-4474-11/2022	EPI3867439	EPI3867440	EPI3867438	EPI3867442	EPI3867435	EPI3867441	EPI3867437	EPI3867436
A/swine/Italy/22-4474-12/2022	EPI3867447	EPI3867448	EPI3867446	EPI3867450	EPI3867443	EPI3867449	EPI3867445	EPI3867444
A/swine/Italy/22-95513-2/2022	EPI3867455	EPI3867456	EPI3867454	EPI3867458	EPI3867451	EPI3867457	EPI3867453	EPI3867452
A/swine/Italy/22-45445-4/2022	EPI3867463	EPI3867464	EPI3867462	EPI3867466	EPI3867459	EPI3867465	EPI3867461	EPI3867460
A/swine/Italy/22-16857-8/2022	EPI3867471	EPI3867472	EPI3867470	EPI3867474	EPI3867467	EPI3867473	EPI3867469	EPI3867468
A/swine/Italy/18-170856-1/2018	EPI3867479	EPI3867480	EPI3867478	EPI3867482	EPI3867475	EPI3867481	EPI3867477	EPI3867476
A/swine/Italy/18-170725/2018	EPI3867487	EPI3867488	EPI3867486	EPI3867490	EPI3867483	EPI3867489	EPI3867485	EPI3867484
A/swine/Italy/21-119840/2021	EPI3867495	EPI3867496	EPI3867494	EPI3867498	EPI3867491	EPI3867497	EPI3867493	EPI3867492
A/swine/Italy/17-66778-2/2017	EPI3867503	EPI3867504	EPI3867502	EPI3867506	EPI3867499	EPI3867505	EPI3867501	EPI3867500

A/swine/Italy/17-66778-3/2017	EPI3867511	EPI3867512	EPI3867510	EPI3867514	EPI3867507	EPI3867513	EPI3867509	EPI3867508
A/swine/Italy/22-20450-4/2022	EPI3867519	EPI3867520	EPI3867518	EPI3867522	EPI3867515	EPI3867521	EPI3867517	EPI3867516
A/swine/Italy/22-23346-6/2022	EPI3867528	EPI3867530	EPI3867526	EPI3867535	EPI3867523	EPI3867531	EPI3867525	EPI3867524
A/swine/Italy/19-34766-3/2019	EPI3867544	EPI3867546	EPI3867542	EPI3867550	EPI3867536	EPI3867547	EPI3867541	EPI3867538
A/swine/Italy/21-69888-5/2021	EPI3867560	EPI3867563	EPI3867558	EPI3867569	EPI3867552	EPI3867566	EPI3867556	EPI3867554
A/swine/Italy/21-81998-1/2021	EPI3867577	EPI3867579	EPI3867575	EPI3867584	EPI3867571	EPI3867581	EPI3867574	EPI3867573
A/swine/Italy/21-69884-5/2021	EPI3867589	EPI3867590	EPI3867588	EPI3867592	EPI3867585	EPI3867591	EPI3867587	EPI3867586
A/swine/Italy/22-77301-10/2022	EPI3867597	EPI3867598	EPI3867596	EPI3867600	EPI3867593	EPI3867599	EPI3867595	EPI3867594
A/swine/Italy/22-21550/2022	EPI3867605	EPI3867606	EPI3867604	EPI3867608	EPI3867601	EPI3867607	EPI3867603	EPI3867602
A/swine/Italy/711/2006	EPI3867613	EPI3867614	EPI3867612	EPI3867616	EPI3867609	EPI3867615	EPI3867611	EPI3867610
A/swine/Italy/22-/95513-1/2022	EPI3867644	EPI3867645	EPI3867643	EPI3867647	EPI3867640	EPI3867646	EPI3867642	EPI3867641
A/swine/Italy/716/2006	EPI3917131	EPI3917132	EPI3917130	EPI3917134	EPI3917127	EPI3917133	EPI3917129	EPI3917128
A/Verona/2810/2009	EPI3917139	EPI3917140	EPI3917138	EPI3917142	EPI3917135	EPI3917141	EPI3917137	EPI3917136
A/swine/Italy/13-139329-1/2013	EPI694895	EPI694896	EPI694894	EPI694898	EPI694891	EPI694897	EPI694893	EPI694892
A/swine/Italy/13-139329-70/2013	EPI694903	EPI694904	EPI694902	EPI694906	EPI694899	EPI694905	EPI694901	EPI694900
A/swine/Italy/4660-3/2009	EPI3918088	EPI3918087	EPI3918086	EPI917483	EPI3918084	EPI3918083	EPI3918082	EPI3918085
A/swine/Italy/13-139329/2013	EPI1917471	EPI1917472	EPI1917470	EPI1917474	EPI1917467	EPI1917473	EPI1917469	EPI1917468
A/swine/Italy/14-76195/2014	EPI1917479	EPI1917480	EPI1917478	EPI1917482	EPI1917475	EPI1917481	EPI1917477	EPI1917476
A/swine/Italy/15-88547-62/2015	EPI1917487	EPI1917488	EPI1917486	EPI1917490	EPI1917483	EPI1917489	EPI1917485	EPI1917484
A/swine/Italy/14-41186/2014	EPI1917495	EPI1917496	EPI1917494	EPI1917498	EPI1917491	EPI1917497	EPI1917493	EPI1917492
A/swine/Italy/13-163032/2013	EPI1917503	EPI1917504	EPI1917502	EPI1917506	EPI1917499	EPI1917505	EPI1917501	EPI1917500
A/swine/Italy/14-123611-6/2014	EPI1917511	EPI1917512	EPI1917510	EPI1917514	EPI1917507	EPI1917513	EPI1917509	EPI1917508
A/swine/Italy/14-30549-02/2014	EPI1917519	EPI1917520	EPI1917518	EPI1917522	EPI1917515	EPI1917521	EPI1917517	EPI1917516
A/swine/Italy/15-133839-26/2015	EPI1917527	EPI1917528	EPI1917526	EPI1917530	EPI1917523	EPI1917529	EPI1917525	EPI1917524
A/swine/Italy/14-99219-4/2014	EPI1917535	EPI1917536	EPI1917534	EPI1917538	EPI1917531	EPI1917537	EPI1917533	EPI1917532
A/swine/Italy/16-28766-15/2016	EPI1917543	EPI1917544	EPI1917542	EPI1917546	EPI1917539	EPI1917545	EPI1917541	EPI1917540
A/swine/Italy/15-114019-6/2015	EPI1917551	EPI1917552	EPI1917550	EPI1917554	EPI1917547	EPI1917553	EPI1917549	EPI1917548
A/swine/Italy/16-53613-6/2016	EPI1917559	EPI1917560	EPI3748347	EPI1917562	EPI1917555	EPI1917561	EPI1917557	EPI1917556
A/swine/Italy/18-45794-40/2018	MZ477559	MZ477579	MZ477577	MZ477562	MZ477568	MZ477570	MZ477565	MZ477574
A/swine/Italy/18-45794-43/2018	MZ477560	MZ541590	MZ477578	MZ477563	MZ477569	MZ477572	MZ477566	MZ477575
A/swine/Italy/18-45794-37/2018	MZ477558	MZ541589	MZ477576	MZ477561	MZ477567	MZ477571	MZ477564	MZ477573

A/swine/Italy/19-142834-25/2019	MZ477664	MZ477662	MZ477660	MZ477650	MZ477656	MZ477654	MZ477652	MZ477658
A/swine/Italy/19-142834-30/2019	MZ477665	MZ477663	MZ477661	MZ477651	MZ477657	MZ477655	MZ477653	MZ477659
A/swine/Italy/19-49885-4/2019	MZ477635	MZ541586	MZ477634	MZ477629	MZ477632	MZ477631	MZ477630	MZ477633
A/swine/Italy/20-17132-10/2020	MZ477614	MZ541584	MZ477613	MZ477608	MZ477611	MZ477610	MZ477609	MZ477612
A/swine/Italy/20-113978-14/2020	MZ477537	MZ477536	MZ477535	MZ477531	MZ477534	MZ477533	MZ477532	EPI2274971
A/swine/Italy/17-24801-21/2017	MZ477543	MZ541582	MZ477542	MZ477538	MZ477540	MZ477539	PV101480	MZ477541
A/swine/Italy/19-162921-14/2019	MZ484034	MZ541574	MZ541573	MZ484039	MZ484036	MZ484037	MZ484038	MZ484035
A/swine/Italy/19-163539-16/2019	MZ484040	MZ541576	MZ541575	MZ484045	MZ484042	MZ484043	MZ484044	MZ484041
A/swine/Italy/18-82500/2018	MZ477587	MZ541592	MZ477593	MZ477588	MZ477591	MZ477590	MZ477589	MZ477592
A/swine/Italy/19-61749-11/2019	MZ477607	MZ541585	MZ477606	MZ477601	MZ477604	MZ477603	MZ477602	MZ477605
A/swine/Italy/19-66854-2/2019	MZ477711	MZ477710	MZ477709	MZ477704	MZ477707	MZ477706	MZ477705	MZ477708
A/swine/Italy/20-18057-6/2020	MZ477719	MZ477718	MZ477717	MZ477712	MZ477715	MZ477714	MZ477713	MZ477716
A/swine/Italy/17-2895/2017	MZ484010	MZ541598	MZ484009	MZ484005	MZ484007	MZ484006	PV101481	MZ484008
A/swine/Italy/18-64849-32/2018	MZ477544	MZ541583	MZ477550	MZ477545	MZ477548	MZ477547	MZ477546	MZ477549
A/swine/Italy/18-82770-28/2018	MZ483990	MZ541600	MZ483989	MZ483984	MZ483987	MZ483986	MZ483985	MZ483988
A/swine/Italy/18-109888-31/2018	MZ477551	MZ541595	MZ477557	MZ477552	MZ477555	MZ477554	MZ477553	MZ477556
A/swine/Italy/17-165437/2017	MZ484051	PV101476	PV101479	MZ484046	MZ484049	MZ484048	MZ484047	MZ484050
A/swine/Italy/19-125153-7/2019	MZ477649	MZ541594	MZ477647	MZ477637	MZ477643	MZ477641	MZ477639	MZ477645
A/swine/Italy/19-125153-19/2019	MZ477648	MZ541593	MZ477646	MZ477636	MZ477642	MZ477640	MZ477638	MZ477644
A/swine/Italy/17-66778-1/2017	MZ484016	MZ541599	MZ484015	MZ484011	MZ484013	MZ484012	PV101489	MZ484014
A/swine/Italy/17-147155-16/2017	MZ477627	MZ541579	MZ477625	MZ477615	MZ477621	MZ477619	MZ477617	MZ477623
A/swine/Italy/17-147155-17/2017	MZ477628	MZ541580	MZ477626	MZ477616	MZ477622	MZ477620	MZ477618	MZ477624
A/swine/Italy/17-66671-3/2017	MZ477695	MZ477694	MZ477693	MZ477688	MZ477691	MZ477690	MZ477689	MZ477692
A/swine/Italy/17-137733-46/2017	MZ484022	MZ541601	PV101478	MZ484017	MZ484020	MZ484019	MZ484018	MZ484021
A/swine/Italy/18-57405-3/2018	MZ477580	MZ541591	MZ477586	MZ477581	MZ477584	MZ477583	MZ477582	MZ477585
A/swine/Italy/19-23998-3/2019	MZ477600	MZ541587	MZ477599	MZ477594	MZ477597	MZ477596	MZ477595	MZ477598
A/swine/Italy/19-162065-4/2019	MZ477680	MZ477687	MZ477686	MZ477681	MZ477684	MZ477683	MZ477682	MZ477685
A/swine/Italy/18-170704/2018	MZ477672	MZ541588	MZ477671	MZ477666	MZ477669	MZ477668	MZ477667	MZ477670
A/swine/Italy/17-55088-13/2017	MZ484004	PV101477	MZ484001	MZ483994	MZ483998	MZ483997	PV101484	PV101486
A/swine/Italy/17-55088-15/2017	MZ484003	MZ484002	MZ484000	MZ483995	MZ483999	MZ483996	PV101487	PV101488
A/swine/Italy/17-34594-3/2017	PV101483	MZ541597	MZ541596	MZ483991	MZ483993	MZ483992	PV101482	PV101485

A/swine/Italy/20-69081-12/2020	MZ484023	MZ541578	MZ541577	MZ484028	MZ484025	MZ484026	MZ484027	MZ484024
A/swine/Italy/19-176305-7/2019	MZ484063	MZ484062	PV133775	MZ484052	MZ484058	MZ484056	MZ484054	MZ484060
A/swine/Italy/19-176304-1/2019	MZ477703	MZ477702	MZ477701	MZ477696	MZ477699	MZ477698	MZ477697	MZ477700
A/swine/Italy/20-5703-2/2020	MZ477679	MZ477678	MZ541581	MZ477673	MZ477676	MZ477675	MZ477674	MZ477677
A/swine/Italy/20-5707-1/2020	MZ477735	MZ477734	MZ477733	MZ477728	MZ477731	MZ477730	MZ477729	MZ477732
A/swine/Italy/20-5709-1/2020	MZ477743	MZ477742	MZ477741	MZ477736	MZ477739	MZ477738	MZ477737	MZ477740
A/swine/Italy/20-30224-4/2020	MZ477720	MZ477727	MZ477726	MZ477721	MZ477724	MZ477723	MZ477722	MZ477725
A/swine/Italy/20-98963-3/2020	MZ484033	MZ541572	MZ484071	MZ542323	MZ484031	MZ484030	MZ484029	MZ484032

Supplementary Table 2. Tested swine farms and samples per year.

Year	Tested Samples	Positive samples	% Positive samples	Farms	Positive Farms	% positive farms
2013	227	31	13.7%	5	3	60.0%
2014	437	56	12.8%	25	5	20.0%
2015	67	15	22.4%	16	4	25.0%
2016	63	16	25.4%	21	5	23.8%
2017	478	28	5.9%	36	14	38.9%
2018	440	47	10.7%	50	10	20.0%
2019	359	101	28.1%	85	26	30.6%
2020	369	110	29.8%	91	28	30.8%
2021	405	104	25.7%	67	20	29.9%
2022	211	71	33.6%	75	29	38.7%
Total	**3056**	**579**	**18.9%**	**471**	**144**	**30.6%**
Total (counted once)	**-**	**-**	**-**	**253**	**96**	**37.9%**

Supplementary Table 3. Tested swine farms according to province and region of origin.

Region	Province	Tested Farms	Positive Farms	% Positive Farms
Trentino Alto Adige	BZ	2	1	50.0%
	TN	2	0	0.0%
	**Total**	**4**	**1**	**25.0%**
Friuli Venezia Giulia	GO	4	2	50.0%
	PN	41	16	39.0%
	UD	32	11	34.4%
	**Total**	**77**	**29**	**37.7%**
Veneto	BL	15	0	0.0%
	PD	37	15	40.5%
	RO	5	1	20.0%
	TV	45	16	35.6%
	VE	9	4	44.4%
	VI	12	3	25.0%
	VR	49	27	55.1%
	**Total**	**172**	**66**	**38.4%**
Total		**253**	**96**	**37.9%**

BZ=Bolzano, TN=Trento, GO=Gorizia, PN=Pordenone, UD=Udine, BL=Belluno, PD=Padova, RO=Rovigo, TV=Treviso, VE=Venezia, VI=Vicenza, VR=Verona.

Supplementary table 4. Sequenced swIAV positive samples in Northeast Italy. Av = Eurasian avian-like H1avN1; pdm = A(H1N1)pdm09; N2g = A/swine/Gent/1/1984-like H3N2; Hu = human-like; N2it = A/swine/Italy/4675/2003-like N2. VEN = Veneto; FVG = Friuli-Venezia Giulia.

Sample name	Farm #	Province	Region	Genotype	Collection date	Subtype	HA	NA	PB2	PB1	PA	NP	NS	MP
A/swine/Italy/13-163032/2013	1	PD	VEN	D	18/12/2013	H1N2	1C.2.5	N2g	Av	Av	Av	Av	Av	Av
A/swine/Italy/15-133839-26/2015	1	PD	VEN	D	01/11/2015	H1N2	1C.2.5	N2g	Av	Av	Av	Av	Av	Av
A/swine/Italy/18-45794-40/2018	1	PD	VEN	D	04/04/2018	H1N2	1C.2.5	N2g	Av	Av	Av	Av	Av	Av
A/swine/Italy/18-45794-43/2018	1	PD	VEN	P	04/04/2018	H1N1	1A.3.3.2	pdm	pdm	pdm	pdm	pdm	pdm	pdm
A/swine/Italy/18-45794-37/2018	1	PD	VEN	P	04/04/2018	H1N1	1A.3.3.2	pdm	pdm	pdm	pdm	pdm	pdm	pdm
A/swine/Italy/19-142834-25/2019	1	PD	VEN	D	30/10/2019	H1N2	1C.2.5	N2g	Av	Av	Av	Av	Av	Av
A/swine/Italy/19-142834-30/2019	1	PD	VEN	D	30/10/2019	H1N2	1C.2.5	N2g	Av	Av	Av	Av	Av	Av
A/swine/Italy/13-139329/2013	2	TV	VEN	Novel2013	31/10/2013	H1N2	1B.1.2.2	N2it	Av	Av	Av	Av	Av	pdm
A/swine/Italy/13-139329-1/2013	2	TV	VEN	Novel2013	31/10/2013	H1N2	1B.1.2.2	N2it	Av	Av	Av	Av	Av	pdm
A/swine/Italy/13-139329-70/2013	2	TV	VEN	Novel2013	31/10/2013	H1N2	1B.1.2.2	N2it	Av	Av	Av	Av	Av	pdm
A/swine/Italy/13-166853-62/2013	2	TV	VEN	F	18/12/2013	H1N2	1B.1.2.2	N2it	Av	Av	Av	Av	Av	Av
A/swine/Italy/13-166853-65/2013	2	TV	VEN	F	27/12/2013	H1N2	1B.1.2.2	N2it	Av	Av	Av	Av	Av	Av
A/swine/Italy/14-3494-2/2014	2	TV	VEN	Novel2013	27/12/2013	H1N2	1B.1.2.2	N2it	Av	Av	Av	Av	Av	pdm
A/swine/Italy/14-3499-21/2014	2	TV	VEN	F	08/01/2014	H1N2	1B.1.2.2	N2it	Av	Av	Av	Av	Av	Av
A/swine/Italy/14-3494-3/2014	2	TV	VEN	Novel2013	08/01/2014	H1N2	1B.1.2.2	N2it	Av	Av	Av	Av	Av	pdm
A/swine/Italy/14-3499-28/2014	2	TV	VEN	Novel2013	29/01/2014	H1N2	1B.1.2.2	N2it	Av	Av	Av	Av	Av	pdm
A/swine/Italy/14-41186/2014	2	TV	VEN	Novel2013	29/01/2014	H1N2	1B.1.2.2	N2it	Av	Av	Av	Av	Av	pdm

A/swine/Italy/14-76195/2014	2	TV	VEN	Novel2013	12/03/2014	H1N2	1B.1.2.2	N2it	Av	Av	Av	Av	Av	pdm
A/swine/Italy/19-49885-4/2019	2	TV	VEN	U	15/04/2019	H1N1	1C.2.1	Av	pdm	pdm	pdm	pdm	pdm	pdm
A/swine/Italy/22-119951-2/2022	2	TV	VEN	Novel 2	16/09/2022	H1N2	1A.3.3.2	N2g	pdm	pdm	pdm	pdm	Av	pdm
A/swine/Italy/16-53613-6/2016	3	PD	VEN	P	03/05/2016	H1N1	1A.3.3.2	pdm	pdm	pdm	pdm	pdm	pdm	pdm
A/swine/Italy/20-17132-10/2020	3	PD	VEN	P	06/02/2020	H1N1	1A.3.3.2	pdm	pdm	pdm	pdm	pdm	pdm	pdm
A/swine/Italy/17-35485-4/2017	4	PD	VEN	B	29/05/2017	H3N2	H3	N2g	Av	Av	Av	Av	Av	Av
A/swine/Italy/20-113978-14/2020	4	PD	VEN	F	18/09/2020	H1N2	1B.1.2.2	N2it	Av	Av	Av	Av	Av	Av
A/swine/Italy/17-24801-21/2017	5	VE	VEN	P	03/01/2017	H1N1	1A.3.3.2	pdm	pdm	pdm	pdm	pdm	pdm	pdm
A/swine/Italy/19-162921-14/2019	5	VE	VEN	T	06/12/2019	H1N2	1C.2.5	N2g	pdm	pdm	pdm	pdm	pdm	pdm
A/swine/Italy/19-163539-16/2019	5	VE	VEN	T	09/12/2019	H1N2	1C.2.5	N2g	pdm	pdm	pdm	pdm	pdm	pdm
A/swine/Italy/18-82500/2018	6	TV	VEN	P	19/06/2018	H1N1	1A.3.3.2	pdm	pdm	pdm	pdm	pdm	pdm	pdm
A/swine/Italy/19-61749-11/2019	6	TV	VEN	P	14/05/2019	H1N1	1A.3.3.2	pdm	pdm	pdm	pdm	pdm	pdm	pdm
A/swine/Italy/19-59120-13/2019	7	PD	VEN	M	07/05/2019	H1N1	1C.2.5	Av	Av	Av	Av	Av	Av	pdm
A/swine/Italy/19-66854-2/2019	7	PD	VEN	M	22/05/2019	H1N1	1C.2.5	Av	Av	Av	Av	Av	Av	pdm
A/swine/Italy/20-18057-6/2020	7	PD	VEN	M	05/02/2020	H1N1	1C.2.5	Av	Av	Av	Av	Av	Av	pdm
A/swine/Italy/17-2895/2017	8	PN	FVG	T	17/03/2017	H1N2	1C.2.4	N2g	pdm	pdm	pdm	pdm	pdm	pdm
A/swine/Italy/18-64849-32/2018	8	PN	FVG	P	10/05/2018	H1N1	1A.3.3.2	pdm	pdm	pdm	pdm	pdm	pdm	pdm
A/swine/Italy/18-82770-28/2018	8	PN	FVG	P	19/06/2018	H1N1	1A.3.3.2	pdm	pdm	pdm	pdm	pdm	pdm	pdm
A/swine/Italy/18-109888-31/2018	8	PN	FVG	P	24/08/2018	H1N1	1A.3.3.2	pdm	pdm	pdm	pdm	pdm	pdm	pdm
A/swine/Italy/17-165437/2017	9	PN	FVG	T	18/12/2017	H1N2	1C.2.4	N2g	pdm	pdm	pdm	pdm	pdm	pdm

A/swine/Italy/19-125153-7/2019	9	PN	FVG	AH	26/09/2019	H1N2	1C.2.4	N2g	pdm	pdm	pdm	pdm	Av	pdm
A/swine/Italy/19-125153-19/2019	9	PN	FVG	AH	26/09/2019	H1N2	1C.2.4	N2g	pdm	pdm	pdm	pdm	Av	pdm
A/swine/Italy/17-66778-1/2017	10	UD	FVG	T	04/05/2017	H1N2	1C.2.4	N2g	pdm	pdm	pdm	pdm	pdm	pdm
A/swine/Italy/17-66778-2/2017	10	UD	FVG	T	09/05/2017	H1N2	1C.2.4	N2g	pdm	pdm	pdm	pdm	pdm	pdm
A/swine/Italy/17-66778-3/2017	10	UD	FVG	T	09/05/2017	H1N2	1C.2.4	N2g	pdm	pdm	pdm	pdm	pdm	pdm
A/swine/Italy/17-147155-16/2017	10	UD	FVG	T	16/11/2017	H1N2	1C.2.4	N2g	pdm	pdm	pdm	pdm	pdm	pdm
A/swine/Italy/17-147155-17/2017	10	UD	FVG	T	16/11/2017	H1N2	1C.2.4	N2g	pdm	pdm	pdm	pdm	pdm	pdm
A/swine/Italy/22-20450-4/2022	10	UD	FVG	Novel 1	14/02/2022	H1N2	1C.2.4	N2g	pdm	Av	pdm	pdm	pdm	pdm
A/swine/Italy/22-23346-6/2022	10	UD	FVG	Novel 1	22/02/2022	H1N2	1C.2.4	N2g	pdm	Av	pdm	pdm	pdm	pdm
A/swine/Italy/14-123611-6/2014		PN	FVG	P	09/10/2014	H1N1	1A.3.3.2	pdm	pdm	pdm	pdm	pdm	pdm	pdm
A/swine/Italy/15-88547-62/2015		PN	FVG	D	01/07/2015	H1N2	1C.2.5	N2g	Av	Av	Av	Av	Av	Av
A/swine/Italy/17-66671-3/2017		PN	FVG	A	29/05/2017	H1N1	1C.2.1	Av	Av	Av	Av	Av	Av	Av
A/swine/Italy/17-137733-46/2017		PN	FVG	T	27/10/2017	H1N2	1C.2.4	N2g	pdm	pdm	pdm	pdm	pdm	pdm
A/swine/Italy/18-57405-3/2018		PN	FVG	P	18/04/2018	H1N1	1A.3.3.2	pdm	pdm	pdm	pdm	pdm	pdm	pdm
A/swine/Italy/19-34766-3/2019		PN	FVG	B	22/03/2019	H3N2	H3	N2g	Av	Av	Av	Av	Av	Av
A/swine/Italy/21-69884-5/2021		PN	FVG	T	27/05/2021	H1N2	1C.2.4	N2g	pdm	pdm	pdm	pdm	pdm	pdm
A/swine/Italy/21-69888-5/2021		PN	FVG	T	31/05/2021	H1N2	1C.2.4	N2g	pdm	pdm	pdm	pdm	pdm	pdm
A/swine/Italy/21-81998-1/2021		PN	FVG	T	24/06/2021	H1N2	1C.2.4	N2g	pdm	pdm	pdm	pdm	pdm	pdm
A/swine/Italy/22-21550/2022		PN	FVG	Novel 1	17/02/2022	H1N2	1C.2.4	N2g	pdm	Av	pdm	pdm	pdm	pdm
A/swine/Italy/14-30549-02/2014		UD	FVG	A	26/06/2014	H1N1	1C.2.1	Av	Av	Av	Av	Av	Av	Av

A/swine/Italy/19-23998-3/2019		UD	FVG	P	21/02/2019	H1N1	1A.3.3.2	pdm	pdm	pdm	pdm	pdm	pdm	pdm
A/swine/Italy/19-162065-4/2019		UD	FVG	D	04/12/2019	H1N2	1C.2.5	N2g	Av	Av	Av	Av	Av	Av
A/swine/Italy/22-77301-10/2022		UD	FVG	A	06/06/2022	H1N1	1C.2.2	Av	Av	Av	Av	Av	Av	Av
A/swine/Italy/14-99219-4/2014		PD	VEN	B	26/08/2014	H3N2	H3	N2g	Av	Av	Av	Av	Av	Av
A/swine/Italy/16-28766-15/2016		PD	VEN	D	01/03/2016	H1N2	1C.2	N2g	Av	Av	Av	Av	Av	Av
A/swine/Italy/17-55088-13/2017		PD	VEN	A	04/05/2017	H1N1	1C.2.5	Av	Av	Av	Av	Av	Av	Av
A/swine/Italy/17-55088-15/2017		PD	VEN	A	04/05/2017	H1N1	1C.2.5	Av	Av	Av	Av	Av	Av	Av
A/swine/Italy/22-52972-1/2022		RO	VEN	T	19/04/2022	H1N2	1C.2.4	N2g	pdm	pdm	pdm	pdm	pdm	pdm
A/swine/Italy/15-114019-6/2015		TV	VEN	D	01/09/2015	H1N2	1C.2.5	N2g	Av	Av	Av	Av	Av	Av
A/swine/Italy/17-34594-3/2017		TV	VEN	AH	24/02/2017	H1N2	1C.2.4	N2g	pdm	pdm	pdm	pdm	Av	pdm
A/swine/Italy/20-69081-12/2020		TV	VEN	T	04/06/2020	H1N2	1C.2.5	N2g	pdm	pdm	pdm	pdm	pdm	pdm
A/swine/Italy/21-91168-5/2021		TV	VEN	T	16/07/2021	H1N2	1C.2.4	N2g	pdm	pdm	pdm	pdm	pdm	pdm
A/swine/Italy/22-45445-4/2022		TV	VEN	A	04/04/2022	H1N1	1C.2.2	Av	Av	Av	Av	Av	Av	Av
A/swine/Italy/22-/95513-1/2022		TV	VEN	A	13/07/2022	H1N1	1C.2.2	Av	Av	Av	Av	Av	Av	Av
A/swine/Italy/22-95513-2/2022		TV	VEN	A	13/07/2022	H1N1	1C.2.2	Av	Av	Av	Av	Av	Av	Av
A/swine/Italy/19-176305-7/2019		VR	VEN	A	19/12/2019	H1N1	1C.2.1	Av	Av	Av	Av	Av	Av	Av
A/swine/Italy/19-176304-1/2019		VR	VEN	A	19/12/2019	H1N1	1C.2.1	Av	Av	Av	Av	Av	Av	Av
A/swine/Italy/20-5703-2/2020		VR	VEN	A	07/01/2020	H1N1	1C.2.1	Av	Av	Av	Av	Av	Av	Av
A/swine/Italy/20-5707-1/2020		VR	VEN	A	07/01/2020	H1N1	1C.2.1	Av	Av	Av	Av	Av	Av	Av
A/swine/Italy/20-5709-1/2020		VR	VEN	A	07/01/2020	H1N1	1C.2.1	Av	Av	Av	Av	Av	Av	Av

A/swine/Italy/20-30224-4/2020		VR	VEN	F	03/03/2020	H1N2	1B.1.2.2	N2it	Av	Av	Av	Av	Av	Av
A/swine/Italy/20-98963-3/2020		VR	VEN	A	11/08/2020	H1N1	1C.2.4	Av	Av	Av	Av	Av	Av	Av
A/swine/Italy/22-4474-11/2022		VR	VEN	A	13/01/2022	H1N1	1C.2.5	Av	Av	Av	Av	Av	Av	Av
A/swine/Italy/22-4474-12/2022		VR	VEN	A	13/01/2022	H1N1	1C.2.5	Av	Av	Av	Av	Av	Av	Av
A/swine/Italy/22-16857-8/2022		VR	VEN	A	08/02/2022	H1N1	1C.2.5	Av	Av	Av	Av	Av	Av	Av
A/swine/Italy/22-28311-3/2022		VR	VEN	A	03/03/2022	H1N1	1C.2.5	Av	Av	Av	Av	Av	Av	Av
A/swine/Italy22-35872-1/2022		VR	VEN	D	17/03/2022	H1N2	1C.2.5	N2g	Av	Av	Av	Av	Av	Av
A/swine/Italy/22-35872-2/2022		VR	VEN	D	17/03/2022	H1N2	1C.2.5	N2g	Av	Av	Av	Av	Av	Av
A/swine/Italy/4159/2006				F	2006	H1N2	1B.1.2.2	N2it	Av	Av	Av	Av	Av	Av
A/swine/Italy/711/2006				A	2006	H1N1	1C.2.1	Av	Av	Av	Av	Av	Av	Av
A/swine/Italy/716/2006				B	2006	H3N2	H3	N2g	Av	Av	Av	Av	Av	Av
A/swine/Italy/4660-3/2009				F	2009	H1N2	1B.1.2.2	N2it	Av	Av	Av	Av	Av	Av
A/Verona/2810/2009				P	2009	H1N1	1A.3.3.2	pdm	pdm	pdm	pdm	pdm	pdm	pdm
